# Delineating modern variation from extinct morphology in the fossil record using shells of the Eastern Box Turtle (*Terrapene carolina*)

**DOI:** 10.1371/journal.pone.0193437

**Published:** 2018-03-07

**Authors:** Natasha S. Vitek

**Affiliations:** Jackson School of Geosciences, The University of Texas at Austin, Austin, Texas, Florida Museum of Natural History and Department of Biology, the University of Florida, Gainesville, Florida, United States of America; Liverpool John Moores University, UNITED KINGDOM

## Abstract

Characterization of morphological variation in the shells of extant Eastern Box Turtles, *Terrapene carolina*, provides a baseline for comparison to fossil populations. It also provides an example of the difficulties inherent to recognizing intraspecific diversity in the fossil record. The degree to which variation in fossils of *T*. *carolina* can be accommodated by extant variation in the species has been disagreed upon for over eighty years. Using morphometric analyses of the carapace, I address the relationship between modern and fossil *T*. *carolina* in terms of sexual dimorphism, geographic and subspecific variation, and allometric variation. Modern *T*. *carolina* display weak male-biased sexual size dimorphism. Sexual shape dimorphism cannot be reliably detected in the fossil record. Rather than a four-part subspecific division, patterns of geographic variation are more consistent with clinal variation between various regions in the species distribution. Allometric patterns are qualitatively similar to those documented in other emydid turtles and explain a significant amount of shape variation. When allometric patterns are accounted for, Holocene specimens are not significantly different from modern specimens. In contrast, several geologically older specimens have significantly different carapace shape with no modern analogue. Those large, fossilized specimens represent extinct variation occupying novel portions of morphospace. This study highlights the need for additional documentation of modern osteological variation that can be used to test hypotheses of intraspecific evolution in the fossil record.

## Introduction

The recognition of extant, intraspecific diversity in the fossil record provides an important body of evidence with which to connect hypotheses of evolution developed from the modern biota to records of evolution on long time scales [1]. However, that connection can be difficult to document. For example, in many species various phylogeographic patterns hindcast dynamic intraspecific evolution in the Pleistocene [2–4]. Where a model hindcasts a species’ presence or absence, the fossil record is already used as a source of evidence to evaluate that model [4]. In contrast, where a model hindcasts the presence of a particular phylogroup or subspecies, current limits on the ability to recognize those groups present a primary challenge to model evaluation [5].

For a limited number of specimens, use of ancient DNA (aDNA) is one potential solution [6,7]. Where applied, it provides evidence corroborating those hypotheses of dynamic turnover in haplotypes and genetic diversity since the last glacial maximum [8–10]. For most specimens, however, aDNA is not recoverable and recognition must be tied to morphology. In addition, aDNA cannot capture diversity due to ecophenotypic plasticity [11].

The morphological identification of intraspecific diversity is frequently framed in terms of subspecies and often relies on soft-tissue morphology such as external coloration or tail length [12–17]. Such information is not preserved in the isolated teeth and bones that make up the majority of the vertebrate fossil record. In that context, closely related species, much less subspecies, are often indistinguishable using current taxonomic diagnoses [18,19].

Quantitative approaches such as geometric morphometrics may allow for the recognition of intraspecific evolution in the isolated skeletal elements found in the fossil record. For example, measurements have been successfully applied to the fossil record of mammalian dental morphology. Those studies recovered patterns of variation that were proposed to reflect extant, intraspecific relationships preserved over thousands to hundreds of thousands of years [1,5,11,20]. They highlight the potential for success of similar approaches in other, non-mammalian vertebrates [1].

An ideal taxon in which to explore this possibility is the Eastern Box Turtle, *Terrapene carolina*. It is a terrestrial, North American turtle with markedly high levels of intraspecific variation and a rich fossil record [21,22]. The turtle is, or was historically, common and widespread throughout the eastern United States as well as parts of eastern Mexico [23,24]. It is currently partitioned into six different subspecies (*T*. *c*. *bauri*, *T*. *c*. *carolina*, *T*. *c*. *major*, *T*. *c*. *mexicana*, *T*. *c*. *triunguis*, and *T*. *c*. *yucatana*), and intergrades among them [22,24,25]. An additional, extinct subspecies or species, *T*. *putnami*, is known from the Pleistocene of North America [26–28]. Five of those seven proposed subspecies have been recognized in the abundant fossil record of *T*. *carolina* [29,30]. However, those identifications are not universally accepted and change from study to study [24,31].

Disagreement about the degree to which the Pleistocene fossil record of *T*. *carolina* reflects the standing variation in modern *T*. *carolina* has been ongoing for over eighty years [21,22,24,29–31]. Without a well-supported connection between the morphology preserved in fossil sites and the modern biota, it is impossible to test the competing hypotheses proposed to explain the evolution of modern genetic and morphological variation in *T*. *carolina* [22,29,32,33]. Quantitative morphological discrimination of three of the extant subspecies was achieved using features of the whole organism [32], but an insufficient number of those features could be applied to the isolated shell elements common to the fossil record of *Terrapene* [22,29].

In order to connect the spatial and temporal record of *T*. *carolina*, use of a single skeletal system as the unit of analysis is necessary. The carapace of *T*. *carolina* is well-suited to this requirement because it acts as a rigid, integrated element in terms of selection, ecological interaction, and taphonomy [34–36]. In particular, the carapacial bones of *T*. *carolina* usually fuse together in adults [25], forming an element that is common enough in the Pleistocene fossil record that several sites contain multiple, complete carapaces [30,37–39].

Using the carapace, the purpose of this study is to address the controversial relationship between modern and fossil *T*. *carolina* through three major goals: (a) quantify the contribution of previously proposed sources of variation, allometric, geographic or subspecific, and sexually dimorphic, to standing variation in the carapaces of modern *T*. *carolina*, (b) determine which, if any sources of variation are sufficiently diagnostic that they could be identified in the fossil record, and (c) evaluate the degree to which variation in fossils of *T*. *carolina* can be accommodated by these sources of variation. This interpretive framework provides little support for the previous subspecies-based interpretations of the Pleistocene fossil record [22,37], but highlights the presence of fossil sites containing complex patterns of variation that have no modern analogue.

### Previous interpretations of the fossil record

The primary, proposed drivers of carapacial variation in modern *T*. *carolina* that have been applied to the fossil record are size and subspecific affinity [22,29,32]. Sexual dimorphism also influences shape and size, but to a relatively smaller, potentially geographically variable degree [32,40]. The taxonomic history of Pleistocene specimens of *T*. *carolina* largely reflects improvements in the understanding of how those factors influence the “exceedingly variable” modern box turtle [21], and their application to the fossil record. The following synopsis is limited to the history of the specimens used in this study but is representative of the Pleistocene record of North American box turtles.

The first included samples to be described in the literature come from Vero, Florida, and were used to establish the species *Terrapene innoxia* [41]. *T*. *innoxia* was described as a small, thin-shelled turtle with a narrow, highly sloped carapace. A large anterior lobe of a plastron was also discovered from the site and described as the type specimen of *Terrapene antipex* [41]. Large specimens were also discovered at the nearby Melbourne locality and described as a new species, *Terrapene singletoni* [42]. In comparison to the previously established, large species *T*. *putnami* and *T*. *canaliculata*, *T*. *singletoni* displayed subtle differences in size, width of the shell and first vertebral, and features of the marginals. Additional specimens were described and used as the basis for synonymizing the large species, *T*. *antipex* and *T*. *canaliculata* [42].

A subsequent series of studies synonymized all purportedly extinct Pleistocene North American box turtle species with the extant *T*. *carolina*. First, *T*. *singletoni*, *T*. *formosa*, and *T*. *innoxia* were synonymized with *T*. *canaliculata* on the basis of the wide range of size and shape variation observed in juvenile and adult *T*. *carolina major* [21]. Then, fossils collected from Friesenhahn Cave and Ingleside were described [38]. Variation within those samples was used as the basis for the further synonymization of the extinct species *T*. *bulverda*, *T*. *impressa*, *T*. *llanensis*, *T*. *marnochi*, *T*. *eurypygia*, and *T*. *whitneyi* with *T*. *canaliculata* [38,43].

As researchers continued to compare the morphology of fossilized specimens with *T*. *carolina*, they came to the conclusion that the variation in the fossil record reflected the subspecific variation seen in the modern biota [22,29]. *Terrapene canaliculata* was eventually synonymized with the remaining species of extinct, large box turtle, *T*. *putnami*, which was in turn shifted to become an extinct subspecies of *T*. *carolina* [29]. Smaller specimens of fossil box turtles from Florida were re-identified as *T*. *c*. *carolina* or *T*. *c*. *bauri*. Smaller specimens from Texas were called *T*. *c*. *triunguis*. Other specimens were re-evaluated and labeled as intergrades of the various subspecies of *T*. *carolina* [22,29,37]. Those identifications were used to derive multiple, conflicting evolutionary histories in which the modern subspecies appeared and intergraded (see [24] for a summary of those evolutionary hypotheses).

Morphological similarity of large box turtles from the Gulf Coast to even larger fossilized carapaces supported the hypothesis that the fossils represented an extension of modern allometric relationships within *T*. *carolina* rather than different species [21,30]. Results of later research supported the hypothesis that some of the largest fossil box turtles referred to *T*. *putnami* may form a sister species to, rather than a subspecies of, *T*. *carolina* [27,28,44]. However, the Pleistocene fossil record of giant box turtles continues to be largely viewed as an extension of modern allometric variation within the species [32].

### Conflicting interpretations of modern variation

Allometric patterns of variation in modern *T*. *carolina* were previously documented in terms of overall shell dimensions [31,45]. As box turtles grow, length increases faster than width, which increases faster than height. A similar pattern is observed in other emydid turtles [36]. However, relationships of particular features of proposed importance in *T*. *carolina*, such as the degree of flaring of different peripherals and the location of highest point of the carapace, have not been quantified and tested for a correlation with size.

Subspecific identifications in the fossil record were based on qualitative characterizations derived from studies of modern box turtles (Table 1, [22,29]). Some quantification of shape formed the basis of those diagnoses, but the quantitative data themselves were unpublished [29,31]. That situation makes it difficult for researchers to evaluate conflicts between published diagnoses and apply them to new fossils [31]. Further complicating previous interpretations of the fossil record, the validity, rank, geographic ranges, and carapace-based morphological diagnoses of the subspecies differ from author to author in recent studies of modern *T*. *carolina* (Table 1) [32,33,46].

**Table 1:** Comparison of osteological features of the carapace considered diagnostic of different subspecies of *Terrapene carolina* by different authors.

	Auffenberg, 1958	Milstead, 1969	Dodd, 2001	Butler et al. 2011
*T*. *c*. *bauri*	Small size	Small-medium size		
		Elongate carapace	Shell narrow. Elongate carapace, sometimes	
	Highly vaulted shell	Highly vaulted carapace		Shell depth
	Highest point of shell posterior to middle of shell	Highest point of shell on third vertebral scute		
		Widest posterior to the bridge		
	Triangular in posterior view	“bulk badly skewed to the rear” (p.45)		
	Less peripheral flaring than *T*. *c*. *major*	Peripherals flared	Peripherals not flared, sometimes	
				Mid-dorsal keel present/absent
*T*. *c*. *carolina*	Not discussed	Relatively small		
		Gently rounded carapace		Shell depth
				Mid-dorsal keel present/absent
*T*. *c*. *major*	Large size	Large size		
		Elongate carapace	Elongate carapace	Not distinctive
	Flattened carapace		High carapace	
			Highest point in the middle of carapace	
	Depression on either side of the mid-dorsal keel, sometimes	Rugose or rounded sagittal section		
		Hump on fifth central scute		
	Posterior peripherals flared	Posterior peripherals flared (“small radius”)		
	Mid-dorsal keel present	Lateral keel above the bridge	Mid-dorsal keel present	
		Flared anterior peripherals	No mid-carapace flaring	
*T*. *c*. *triunguis*	Not discussed	Carapace length shorter than *T*. *c*. *major*, *T*. *c*. *mexicana*, *T*. *c*. *putnami*, and *T*. *c*. *yucatana*		
		Elongate carapace	Narrow carapace	
		Carapace vaulted, both anteriorly and posteriorly	Highest point of the carapace most posterior of any subspecies	Shell depth
		Hump on third central scute		
			Posterior peripherals flared, moderately	
			Mid-dorsal keel present	Mid-dorsal keel present/absent

The use of subspecies as units of analysis, as has been done in *T*. *carolina*, is not without controversy itself. Before variation can be studied in a subspecific context, the concept of a subspecies has to be clarified. That concept and its application is contentious, complex, and has a long history that is not yet finished [47–49]. The co-option of the term “subspecies” to indicate chronospecies, metataxa, or a segment of a lineage in time, makes the concept even more complex in paleontology [50,51]. That history and controversy is reviewed elsewhere [49,52,53]. In short, subspecies are alternatively defined as local adaptations, evolutionary potential, partially independent lineages, or temporarily independent lineages [48,49,54–67]. No single definition can serve as the intersection of those divergent concepts. All can be recognized by a pattern of metapopulations with heritable phenotypes that are particular to a discrete, local environment to the explicit exclusion of a gradated environment. However, multiple processes can produce particular, local phenotypes in a metapopulation. The conflation of those processes into a single term that recognizes a common pattern may contribute to the subspecies controversy [68].

Given that subspecies can potentially exist in the face of gene flow to and from other metapopulations at high or low levels depending on the concept used, subspecies are not appropriate proxies or units of analyses for biological studies [69–71]. Not only are subspecies not necessarily genetically independent, they may be evolutionarily nested in one another or part of any number of other biologically complex scenarios [48,58,59,72,73]. Therefore, treating each subspecies as a discrete entity comparable to all other subspecies [74–76] may not be an accurate portrayal of biological reality. Patterns and levels of variation can be understood more accurately and precisely by studying the variation itself, rather than *a priori* binning variation into subspecific proxies [77–80].

Here, subspecific units are studied in order to compare results with previously published hypotheses, but the primary study of geographic variation is in a spatially explicit context that does not bin specimens *a priori*. If results of spatial analyses support the delimitation of discrete boundaries between significantly different carapace shapes across the landscape, then geographic groups can be used as units of analysis and potentially identified in the fossil record. If not, then it is not appropriate to apply a subspecies framework to the carapacial fossil record of *T*. *carolina*. Those subspecies may still exist, but their recognition in fossils will have to rely on a different morphological system.

Institutional Abbreviations are as follows: **AMNH**—American Museum of Natural History, New York, New York; **CM**—Carnegie Museum, Pittsburgh, Pennsylvania; **KU**—University of Kansas Natural History Museum, Lawrence, Kansas; **NCSM**—North Carolina State Museum, Raleigh, North Carolina; **OMNH**—University of Oklahoma, Sam Noble Oklahoma Museum of Natural History, Norman, Oklahoma; **SCSM**—South Carolina State Museum, Columbia, South Carolina; **TMM**—Texas Memorial Museum, Vertebrate Paleontology Laboratory, The University of Austin, Austin, Texas; **UF**—The University of Florida, Florida State Museum, Gainesville, Florida; **UFea**—Environmental Archaeology Laboratory at the Florida Museum of Natural History; **UF/FGS**—University of Florida/Florida Geological Survey, Gainesville, Florida; **USNM**—National Museum of Natural History, Smithsonian Institution, Washington, D.C.; **YPM-HERR**—Yale University, Peabody Museum of Natural History, New Haven, Connecticut.

## Materials & methods

### Samples

I measured modern (N = 435) and fossilized (N = 57) specimens of *T*. *carolina* from museum collections. The two proposed subspecies of *T*. *carolina* found in Mexico are considered distinct species by some and it may not be appropriate to include them in an analysis of *T*. *carolina* [81]. In practical terms, the number of specimens of *T*. *c*. *mexicana* and *T*. *c*. *yucatana* in accessible collections was too small to warrant the inclusion of those subspecies in this study. That absence and the disagreement over the relationships of the Mexican species or subspecies to the rest of *T*. *carolina* highlights the need for further collection and investigation of box turtles in Mexico. For those reasons, this study is limited to the diversity within *T*. *carolina* on the continental United States. Specimen numbers for each dataset are reported in the S1 and S2 Files.

#### Proxies for maturity

Even in species with indeterminate growth such as *T*. *carolina*, the degree to which growth explains variation in form changes between reproductively mature and immature subsets of a population [82,83]. After reproductive maturity, growth slows and plays a smaller role in structuring morphological variation than it does prior to reproductive maturity [84]. In order not to conflate those two different growth-age relationships in my analyses of size and shape, I looked for a suite of reliable proxies for reproductive maturity that I could apply to both modern turtles and fossilized carapaces. Data from 215 modern specimens of *T*. *carolina* were studied in order to determine if different morphological proxies for maturity provided concordant signals. Those signals could be used as criteria for constructing downstream datasets.

Number of major growth rings (MGR), carapace length, and shell closure have all been proposed or used as proxies for maturity in turtles [85]. For each specimen possible, I counted the number of MGR on the third vertebral scute. Straight-line carapace length was measured using digital calipers [86,87]. I measured shell closure by giving each specimen one of the following ossification scores: (1) open fontanelles within the carapace; (2) no open fontanelles within the carapace, all sutures visible; (3) carapace partially fused, some sutures no longer visible; (4) carapace completely fused, no visible sutures.

Relationships between the three proxies were tested in two ways. First, I calculated the strength (R^2^) and significance (p) of a linear model in which the number of MGR was treated as the independent variable and log-transformed carapace length the dependent. Second, the relationship between carapace ossification or length and number of MGR was tested via two one-way ANOVAs, each using carapace ossification as the independent variable. Pairwise student’s t-tests were used to analyze differences between MGR and carapace length between each ossification class. P values of multiple t-tests were adjusted using the false discovery rate correction [88].

If the relationships between the three variables all changed at approximately the same values for each variable pair, then each variable was considered a faithful proxy for the same underlying phenomenon, sexual maturity. The values at which the relationship changed were taken as minimum criteria that a specimen had to have in order to be included in downstream datasets.

#### Modern morphometric datasets

Two morphometric datasets of modern specimens of *T*. *carolina* were constructed, a larger dataset intended to document patterns of geographic and allometric variation (N = 200), and a smaller dataset intended to document patterns of sexual dimorphism (N = 60). The datasets needed to be constructed and analyzed separately because sexual dimorphism could not be analyzed in the larger dataset. Fewer than 30% of the specimens chosen for studying geographic variation were identified to sex in museum collection records.

It was possible that secondary sexual characters could be used to identify the sex of an osteological or fossilized specimen in the absence of metadata. A concave indentation in the posterior section of the plastron is a secondary sexual characteristic of *T*. *carolina*, but it is not always a reliable indicator [40,84,89]. Before using the presence or absence of a plastral concavity to sex specimens in the larger dataset, it was necessary to evaluate the accuracy of that method using the smaller dataset.

**Fig 1:**
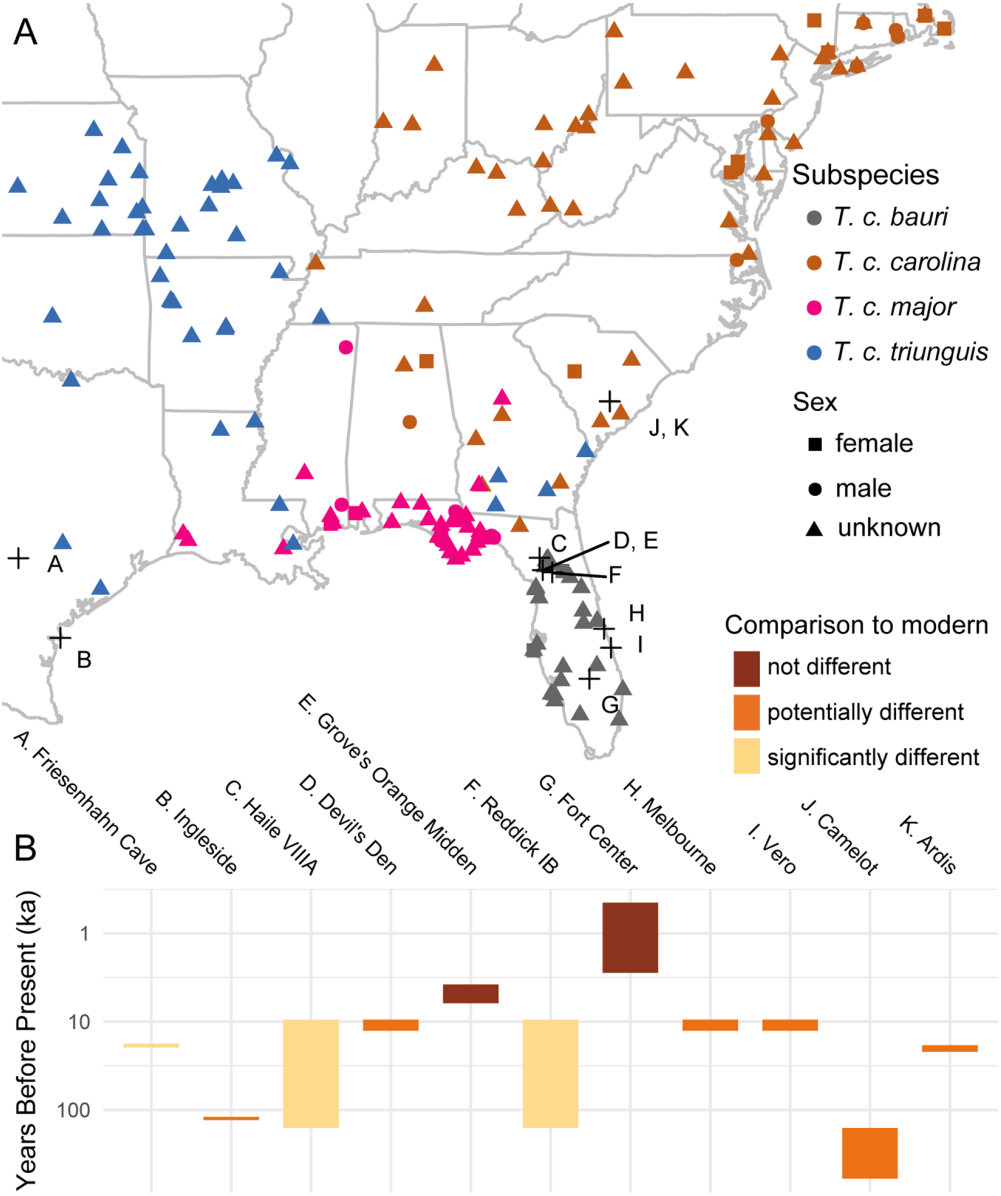
Spatiotemporal extent of sampling of *Terrapene carolina* in this study. (A) Locations from which modern and fossil specimens were collected. Modern specimens included in sexual dimorphism and geographic variation datasets indicated by colored points. Fossil sites indicated by black crosses. Letters correspond to B. (B) Temporal extent of each fossil site, colored by how different morphology at the site was from modern specimens, determined as part of this study.

For three of the subspecies for which adequate numbers of specimens with recorded sex data were available in museum catalogue records (*T*. *c*. *bauri* N = 18, *T*. *c*. *carolina* N = 20, *T*. *c major* N = 14), I collected data from an equal number of males and females each to construct the smaller, sexual dimorphism-oriented dataset (N = 52; Fig 1). In addition to morphometric analyses, the dataset was used to evaluate the accuracy of using the presence or absence of a plastral cavity to sex a specimen in the absence of soft tissue. In order to make allowances for human observational error as opposed to biological variation in secondary sexual characteristics, I qualitatively identified the sex of the individual based on whether or not I could see a concave depression in the posterior part of the plastron in two separate, consecutive rounds. Plastron shape was only considered inaccurate if both rounds resulted in the same incorrect identification of a given specimen and *post hoc* comparison of the collections records could not be reconciled with observed plastron indentation or lack thereof. Because of significant error associated with using this method of sex identification and the small amount of size and shape variation explained by sexual dimorphism, sexes were pooled in the larger dataset (N = 200).

In the larger dataset (N = 200), each of the four subspecies recognized in the United States was represented by 50 specimens sampled from across its geographic range (Fig 1). That sample size should be adequate for recovering potentially biologically significant differences between subspecies, if present, based on previous sensitivity analyses [90]. Both alcohol-preserved and skeletonized specimens were included in the dataset. Where available, latitude and longitude data were taken from specimen records. Where those coordinates were unavailable, specimens were georeferenced using the locality data in collections records. Specimens were chosen as representatives of *T*. *c*. *carolina*, *T*. *c*. *bauri*, *T*. *c*. *major*, and *T*. *c*. *triunguis* if they were identified to the subspecific level in museum records or if they were collected from localities that fell well within multiple published ranges of a given subspecies and outside of intergrade zones (Map 1 of [24], Fig 1 of [32]). Based on the studies of proxies for maturity, specimens were considered suitable for inclusion if their carapace had eight or more growth rings or if all carapacial fontanelles were closed by sutures.

In both datasets, I collected straight line carapace length measurements, counted MGR on the third pleural scute where applicable, evaluated carapace ossification where applicable, and photographed the carapace in dorsal, lateral, and posterior views.

#### Fossil sites and specimens

I collected data from 57 fossilized carapaces from across 10 Pleistocene sites and two Holocene sites (Fig 1). Only localities containing multiple complete, undeformed carapaces were chosen for study in order to obtain an approximation of levels of variation within a given temporal and geographic limit. Lone fossilized shells cannot be identified to species because of high intraspecific variability even in apomorphic characters of *T*. *carolina*, but multiple specimens can be used to contextualize variation [91]. The Holocene sites were pooled together in analyses in order to meet this sample size criterion, though they are figured separately. Specimens studied here have apomorphies for *T*. *carolina* and lack apomorphies for the other three extant species of *Terrapene* [91,92]. Based on the studies of proxies for maturity, specimens were considered suitable for inclusion if all carapacial fontanelles were closed by sutures. Photographs of dorsal, lateral, and posterior views of the carapace were taken for digitization and morphometric analysis. In addition, straight-line carapace lengths were measured with a pair of digital calipers. Due to specimen damage, the following specimens could not be digitized in certain views: UF 5700 (Reddick 1B) could not be digitized in lateral and posterior view, USNM 11834 (Melbourne) could not be digitized in dorsal view, and TMM 933–3039 (Friesenhahn) could not be digitized in dorsal view.

The age of each site was determined based on a review of the published literature. Ages used in this study are differ in some cases from previously published ages (Fig 1, Table 2). More details are provided in the S3 File.

**Table 2:** Location, biostratigraphic age to epoch or North American Land Mammal Age (NALMA), and chronostratigraphic age of each fossil site used in this study, organized by minimum age.

Site	State	Biostratigraphic Age	Age (ka)	References
Fort Center	Florida	Holocene	0.45–2.8	[93,94]
Grove’s Orange Midden	Florida	Holocene	3.8–6.2	[95]
Vero	Florida	Rancholabrean	9.5–12.7	[96–99]
Melbourne	Florida	Rancholabrean	9.5–12.7	[96–99]
Devil’s Den	Florida	Rancholabrean	9.5–12.7	[99,100]
Haile 8A	Florida	Rancholabrean	9.5–160	[37,99,101–105]
Reddick 1B	Florida	Rancholabrean	9.5–160	[99,103–106]
Friesenhahn Cave	Texas	Rancholabrean	17.8–19.6	[38,107]
Ardis	South Carolina	Rancholabrean	18.5–22	[108]
Ingleside	Texas	Rancholabrean	120–130	[99,109–116]
Camelot	South Carolina	Irvingtonian	160–600	[99,117,118]

### Geometric morphometrics

**Fig 2:**
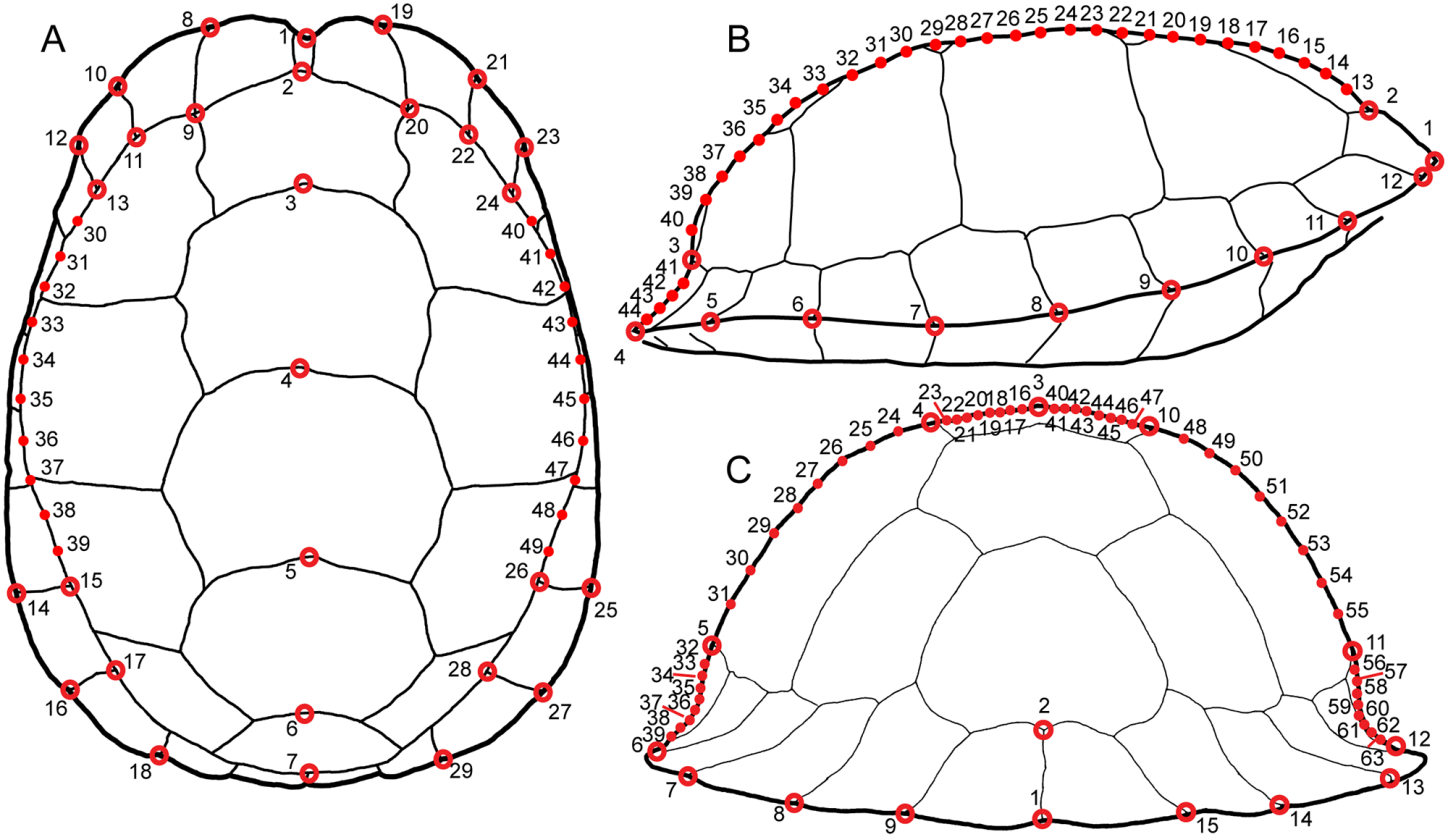
Diagram of landmarks and semilandmarks used to study the shape of the carapace of *Terrapene carolina*. Open circles represent landmarks. Smaller, closed circles represent semilandmarks. (A) Dorsal view. Carapace drawn based on UF 21176. (B) Lateral view. (C) Posterior view. Carapace in B and C drawn based on UF 151664.

In order to adequately analyze a three-dimensional turtle shell using two-dimensional geometric morphometrics, I digitized dorsal, lateral, and posterior views of the carapace (Fig 2, Table 3). Only points that were visible on all complete, undeformed shells, regardless of scute presence/absence or scute morphological variation, were considered as potential landmarks. Because most adult individuals of *T*. *carolina* have fused shells obscuring sutures between bones [24,92], landmarks were placed at the intersections of scutes and on the sulci between them, not on bones and sutures. Semilandmarks were added in order to capture curvature of the carapace that could not be described by landmarks. They were defined as equally distant points between corresponding landmarks.

**Table 3:** Definition of landmark and semilandmark placement.

View	Landmark	Location
Dorsal	1	Anterior-most point on midline
	2–6	Between adjacent scutes along midline
	7	Posterior-most point on midline of vertebral 5
	8–29	Medial and lateral limits of sulci between marginals 1–2, 2–3, 3–4, 8–9; lateral limits of sulci between marginals 7–8, 9–10
	30–49	Semilandmarks: Pleural outline between LM 13 and 15, 24 and 26
Lateral	1	Anterior-most point of carapace
	2	Anterodorsal-most point between nuchal scute and vertebral 1
	3	Posterodorsal-most point between vertebral 5 and marginals
	4	Posterior-most point of carapace
	5–12	ventral-most points on the sulci between marginal 3–11
	13–40	Semilandmarks: Carapace outline between LM 2 and 3
	41–44	Semilandmarks: Carapace outline between LM 3 and 4
Posterior	1–2	Dorsal and lateral limits of sulcus between marginal 12
	3	Dorsal-most point of carapace
	4, 10	Dorsolateral limits between vertebral 3 and adjacent pleurals
	5, 11	Lateral limits between pleurals and marginal
	6–9, 12–15	Posterior limits of sulci between marginals 8–12
	16–23, 40–47	Semilandmarks: Carapace outline between LM 3 and 4, 3 and 10
	24–31, 48–55	Semilandmarks: Carapace outline between LM 4 and 5, 10 and 11
	32–39, 56–63	Semilandmarks: Carapace outline between LM 5 and 6, 11 and 12

LM = landmarks.

Specimens were digitized using tpsDig2.16 [119]. I computed centroid size, or the square root of the sum of squared distances of landmarks from the centroid, then fitted semilandmarks and performed generalized Procrustes superposition to produce sets of aligned coordinates [120] using tpsRelw1.49 [121]. Minimization of Procrustes distance was used as the criterion for sliding semilandmarks. Data were reformatted in R using custom scripts reposited with other analytical code. The position of missing landmarks in symmetrical views of the shell were estimated using OSymm [122]. In order to avoid statistical problems of inflated degrees of freedom associated with analysis of symmetrical objects [123], I used MorphoJ [124] to reflect symmetrical landmarks and used only the symmetrical component of one half of shells including midline landmarks for further analyses of symmetrical views. Although only one half of the shell is analyzed, this procedure uses information from both sides of the specimen and avoids the information loss associated with digitizing only half of a specimen [125]. Principal component (PC) scores were also calculated for each specimen in a principal components analysis (PCA) for each view [126] in R 3.3.3 [127].

Error due to digitization and landmark placement was measured by photographing three arbitrarily chosen specimens three times in each view, then repeatedly placing landmarks five times on an additional arbitrarily chosen fossilized specimen. The identity of the four repeated specimens was used as a variable in a one-way Procrustes ANOVA, which was then used to calculate percent measurement error as well as the intraclass correlation coefficient as a measure of repeatability [128–130]. Measurement error was 8.7% in dorsal view, 3.1% in lateral view and 1.4% in posterior view. Total-dataset repeatability was 0.91, 0.96, and 0.98, respectively. Repeatability was also calculated for each PC derived from the PCA of the complete dataset in each view. In order to reduce dimensionality and limit the impact of measurement error, particularly in canonical variates analyses (CVA), only the repeatable first PCs (dorsal view = 1–5, lateral view = 1–9, posterior view = 1–13) were used in analyses based on PCs [128]. Repeatability values above 0.9 were considered acceptable for the purposes of this study.

### Analyses

Analyses were conducted in R 3.3.3 [127] except where indicated. Graphs and visualizations were composed with the help of the *ggplot2*, *ggmap*, *scales*, *maps*, *plotrix*, *RColorBrewer*, and *wesanderson* packages [131–137]. P values of multiple tests were adjusted using the false discovery rate correction [88]. R scripts are available on GitHub, repository name ‘box-turtle-variation’ (https://github.com/nsvitek/box-turtle-variation). A snapshot of the scripts (DOI: 10.6084/m9.figshare.5768352), along with the raw.tps files (DOI: 10.6084/m9.figshare.5733954), aligned coordinates and metadata (DOI: 10.6084/m9.figshare.5734104) necessary to replicate the study, are archived on FigShare.

#### Sexual dimorphism

Sexual dimorphism was analyzed in terms of both size and shape. Carapace length was used instead of centroid size to measure sexual size dimorphism for the purposes of comparison with previously published literature [138]. In other contexts where landmarks, or shape, were the subjects of an analysis, centroid size was used as a measure of size. Centroid size is generally considered a more appropriate metric of overall size in the context of geometric morphometrics because it is statistically independent of an object’s shape as captured by the configuration of landmarks. However, its use in measuring sexual size dimorphism would unnecessarily limit the ability to compare results to previous findings [139,140]. Centroid size and carapace length were log-transformed, except in the case of calculating sexual dimorphism indices (SDIs) where formulae are intended for untransformed measurements [86,87,138,140].

To measure sexual size dimorphism, first a student’s T-test was used to test for differences in log-transformed carapace length between males and females. Then, sexual size dimorphism was quantified using the “compressed” formulae of Gibbons and Lovich [138]. Although the “uncompressed” formulae were used in a previous study of *T*. *carolina* [141], the compressed formulae are more conducive to modelling and visualization because they are symmetric around zero [138]. Previously reported measures of male and female carapace length were converted to compressed SDI values. Confidence intervals around SDI measurements were calculated through bootstrap resampling [142].

Sexual shape dimorphism was evaluated through both a Procrustes ANOVA of Procrustes superimposed coordinates using the *geomorph* package [143]. In those models, centroid size was included as a covariate to account for the sexual size dimorphism observed in earlier analyses and subspecies identity was included as a covariate to allow for the possibility of different patterns of dimorphism between the groups. In order to evaluate if the resulting patterns of statistically significant sexual shape dimorphism could be used to discriminate the two sexes, datasets of the repeatable PCs in each view were used in jackknife validated assignments tests conducted in CVAGen7b [144]. The assignments test first performs a canonical variates analysis of shape and assigns all specimens to a group based on Mahalanobis distances. Validity of the groups is determined by jackknife assignments in which each specimen is left out of a canonical variates analysis (CVA), then assigned to a group using the CVA axes resulting from the remaining specimens [144]. If the effect of sexual shape dimorphism was small enough that sex could not be accurately assigned by carapace shape alone, then sexes were pooled in downstream analyses.

#### Subspecies

Differences due to subspecific affinity were also measured in terms of both size and shape. The subspecies are characterized in published literature by different mean carapace lengths (Table 1) [22,24,29]. In order to place results into the context of previous research, mean carapace length was compared for each recognized subspecies using ANOVA. Centroid size was also used to compare subspecies in each view using ANOVA.

The degree to which subspecific identity structured variation in carapace shape was explored in four ways. First, the Procrustes superimposed coordinates were subjected to PCA. The first two PCs were plotted to visually inspect to what degree the subspecies occupied distinct regions of morphospace along those primary axes of variation. Second, similar to analyses of sexual dimorphism, datasets of the repeatable PCs in each view were used in jackknife validated assignments tests to test whether or not subspecies could be reliably discriminated based on carapace shape alone. Third, in order to see if groups could be detected within the sample without *a priori* specifying their existence, model-based *k-*means clustering was applied to the dataset [54,145,146]. The approach uses maximum likelihood to fit clustering models to the data, then the Bayesian Information Criterion (BIC) to select the best model including *k*, the number of clusters. The number of potential clusters was searched from *k* = 1 to *k* = 4 in order to allow for the possibility of recognizing all four extant subspecies in the sample.

Fourth, the significance of subspecific identity in explaining shape variation was evaluated through a Procrustes ANOVA of Procrustes superimposed coordinates in each view. Centroid size was included as a covariate to account for documented differences in mean size of different subspecies. In addition, it was necessary to account for the possibility of spatial autocorrelation or clinal variation. Subspecies should be distinct groups, not end members of a broad cline, but the two patterns can be conflated if spatial variation isn’t taken into account [147]. Calculation of Moran’s I for components of both size and shape using the *spdep* package [148] returned significant positive values (data not shown), supporting the hypothesis that significant spatial autocorrelation existed in the dataset. In order account for this spatial autocorrelation, spatial eigenvector mapping (SEVM) was used. SEVM summarizes the major patterns of spatial variation between specimen localities in a limited number of uncorrelated variables [149,150]. A spatial distance matrix between localities was constructed using the *geosphere* package, then spatial eigenvectors (SEs) were calculated using the *vegan* package based on singular value decomposition of that distance matrix [151,152]. In order to limit model complexity, only the two SEs that explained the greatest amount of shape variation were retained for downstream analyses. SEs were chosen using a forward-selection procedure performed with the repeatable PCs of shape variation using the *packfor* package [153,154]. To avoid overfitting, selected SEs were included as covariates one at a time in the Procrustes ANOVA. Only models including SEs that played a significant role in explaining subspecific variation are reported.

#### Fossils

Fossilized specimens were analyzed first by plotting the distribution of carapace lengths to examine whether any site contained a nonoverlapping, bimodal distribution of sizes that would indicate the presence of multiple, sympatric morphs as was proposed for sites like Vero and Melbourne [29]. If bimodality was present at a site, I tested the possibility that sexual size dimorphism could explain the size variation. Given the strong size bimodality of the sites in question, the alternative hypothesis was that the size differences were too great to be explained by sexual size dimorphism. The two groups at a given site would be modelled as males and females and used to calculate an SDI for the site assuming the direction of dimorphism present in the modern species. If that calculated SDI fell outside the 95% confidence interval estimated for SDI of modern *T*. *carolina*, the hypothesis of the two size classes representing two sexes of a single morph was rejected. The two morphs were subsequently analyzed separately.

Two competing, previously published hypotheses of size needed to be addressed before other hypotheses of shape variation could be studied. The first is that large specimens in the fossil record represent an extension of the allometric trajectory present in modern specimens [30]. The second is that large specimens represent a distinct evolutionary unit, whether a species, subspecies, or metataxon, from the modern turtles and that their shape is not simply a reflection of more growth along the same allometric trajectory [27,44]. The hypotheses were addressed using a comparison of slopes. The modern subspecies (N = 200) dataset and the fossil dataset were each pooled and their relationship with centroid size compared using Procrustes ANOVA. Nonsignificant results of previous tests for interactions between centroid size and subspecies and sex, respectively, supported the use of a single allometric model in the modern sample. If the first hypothesis is correct, then the two groups should not have significantly different allometric slopes. In that case, modern and fossilized specimens can be analyzed together with a single model accounting for size. If the second hypothesis is correct, the two groups should have significantly different allometric slopes because “size” in the fossil record contains information about both growth and evolution. In that case, the fossil sample was corrected for allometric growth, but size was not otherwise included as a factor in model comparison. To make this correction, the allometric model for the modern dataset was treated as a model for growth. Given that the modern allometric model accounts for size within the species as well as between its subspecies, it is reasonable to use it as a model for shape differences due to growth as opposed to shape differences due to genetic or evolutionary backgrounds. Both modern and fossil samples were regressed against the modern allometric model. Residual shapes were used as allometrically corrected samples in downstream analyses.

Next, primary patterns of variation were explored using PCA. The presence of discrete groups in the fossil record was tested for using model-based *k-*means clustering using the same parameters as the modern subspecies (N = 200) dataset.

Procrustes ANOVA and disparity were both used to address a fundamental question underlying the interpretation of the fossil record of *T*. *carolina*: can the record as published to date be adequately explained as a reflection of extant variation within the species, or is some of the morphological variation in the fossil record no longer reflected in the modern biota [22,30,32]? First, the shape of each site or morph within a site in the fossil record was compared to the modern sample in a pairwise comparison using Procrustes ANOVA. Next, disparity, a measure of how much morphospace is occupied by a sample, was quantified as the Euclidean distance from all specimens in a sample to the sample’s mean location in principal component space [155,156]. Distances were limited to the repeatable PCs [139]. Use of all PCs did not qualitatively change results.

A simple comparison of the amount of morphospace occupied by the two samples would not answer the question. The fossil sample could occupy an amount of morphospace equal to the modern sample either because (a) the two samples occupy the same morphospace or (b) because the fossil sample occupies an equally large, but only partially overlapping area of morphospace. Instead, the question was statistically framed as whether or not the fossil sample added significant amounts of morphospace when combined with the modern sample. If so, then the fossil record contained novel morphology either not sampled or extirpated from the modern biota.

Before evaluating the contribution of fossilized specimens to overall disparity, I used bootstrapping to test whether a change sample size could account for potential changes in disparity estimates [142]. The larger dataset of modern *T*. *carolina* was resampled with replacement 1000 times for sample sizes ranging from N = 10 to N = 200, increasing sequentially by 10. For each of those 1000 pseudoreplicates, I calculated total disparity. If mean disparity stabilized at a sample size less than N = 200, then any increase in disparity that might occur after adding fossils to the dataset was unlikely to be due to increase in sample size alone. After determining at what point estimates of estimates of disparity were insensitive to sample size, I added fossilized specimens to the full, N = 200 dataset one site or morph at a time, then recalculated disparity. Finally, all 57 fossilized specimens were added to the N = 200 dataset of modern specimens and disparity recalculated. A one-tailed 95% confidence interval was calculated from the distribution of modern specimens at each sample size.

## Results

### Proxies for maturity

**Fig 3:**
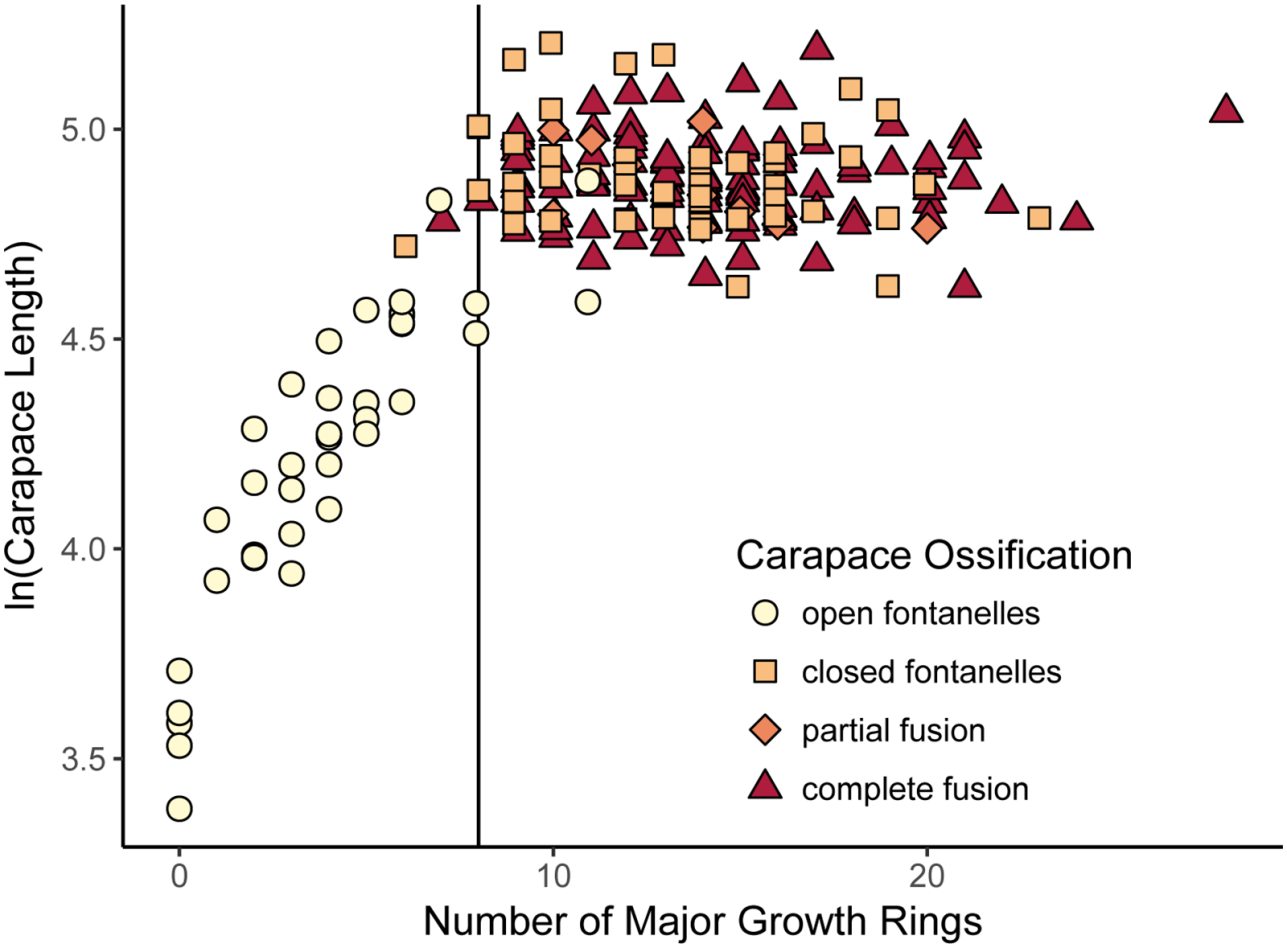
Relationship between three proposed proxies for maturity in *T*. *carolina*. Vertical line indicates the cutoff for number of major growth rings (MGR) used this study as a proxy for attainment of reproductive maturity and its correspondence to degree of carapace ossification.

Carapace length and number of MGR were strongly correlated in specimens with 0–8 growth rings (p < 0.00001, R^2^ = 0.853; Fig 3). When only longer specimens or those with more growth rings were analyzed, there was no correlation between the two variables (p = 0.582, R^2^ = -0.00392). Below 8 MGR and 99 mm carapace length, all carapaces had open fontanelles. At or above 8 MGR and 99 mm, only 0.57% of carapaces had open fontanelles. Two carapaces with fewer than 8 MGR had closed shells, and two carapaces longer than 99 mm had open fontanelles.

Ossification score had a significant relationship with carapace length and number of MGR (length: F(1,214) = 117.69, p = 4.09 x 10^−22^; MGR: F(1,214) = 118.37, p = 3.29 x 10^−22^). Pairwise significant differences were found only between open fontanelles and other ossification stages (p < 0.0001). No significant difference in carapace length or number of growth rings was found between carapaces with closed fontanelles, partially fused carapaces, or fully fused carapaces. Based on the convergence of carapace closure and the loss of a relationship between shell size and number of growth rings, shell closure was considered an additional proxy for attainment of sexual maturity in addition to the presence of eight or more MGR.

### Sexual dimorphism

**Fig 4:**
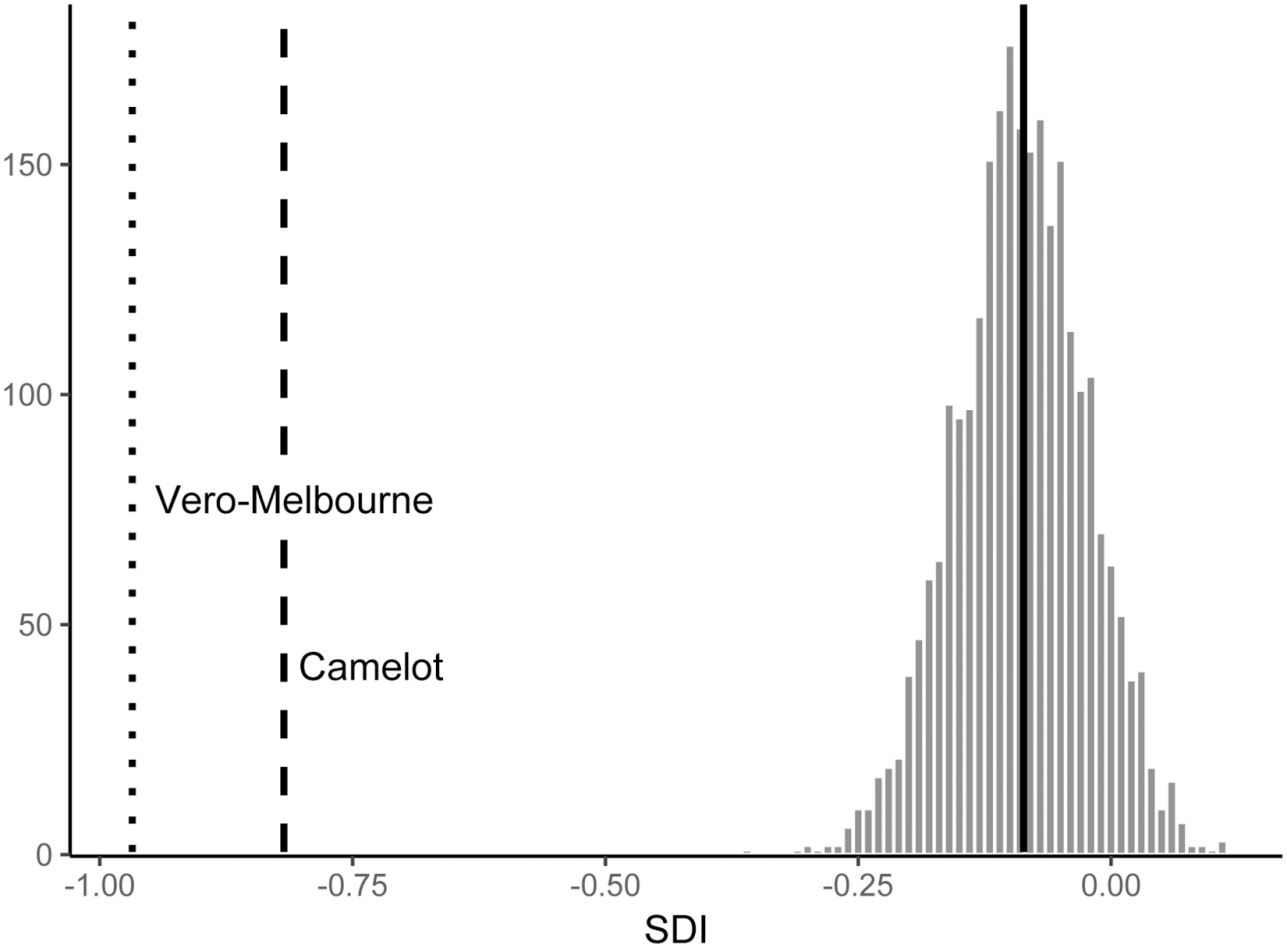
Estimates of sexual size dimorphism in *T*. *carolina* using the compressed sexual dimorphism index (SDI) of Gibbons and Lovich [138]. Heavy solid lines indicate the mean SDI of all specimens in the dataset (N = 60). Black, dashed lines show the results of modelling bimodal distribution of carapace length within a fossil site (Camelot) or site pair (Vero and Melbourne) as sexual dimorphism.

**Fig 5:**
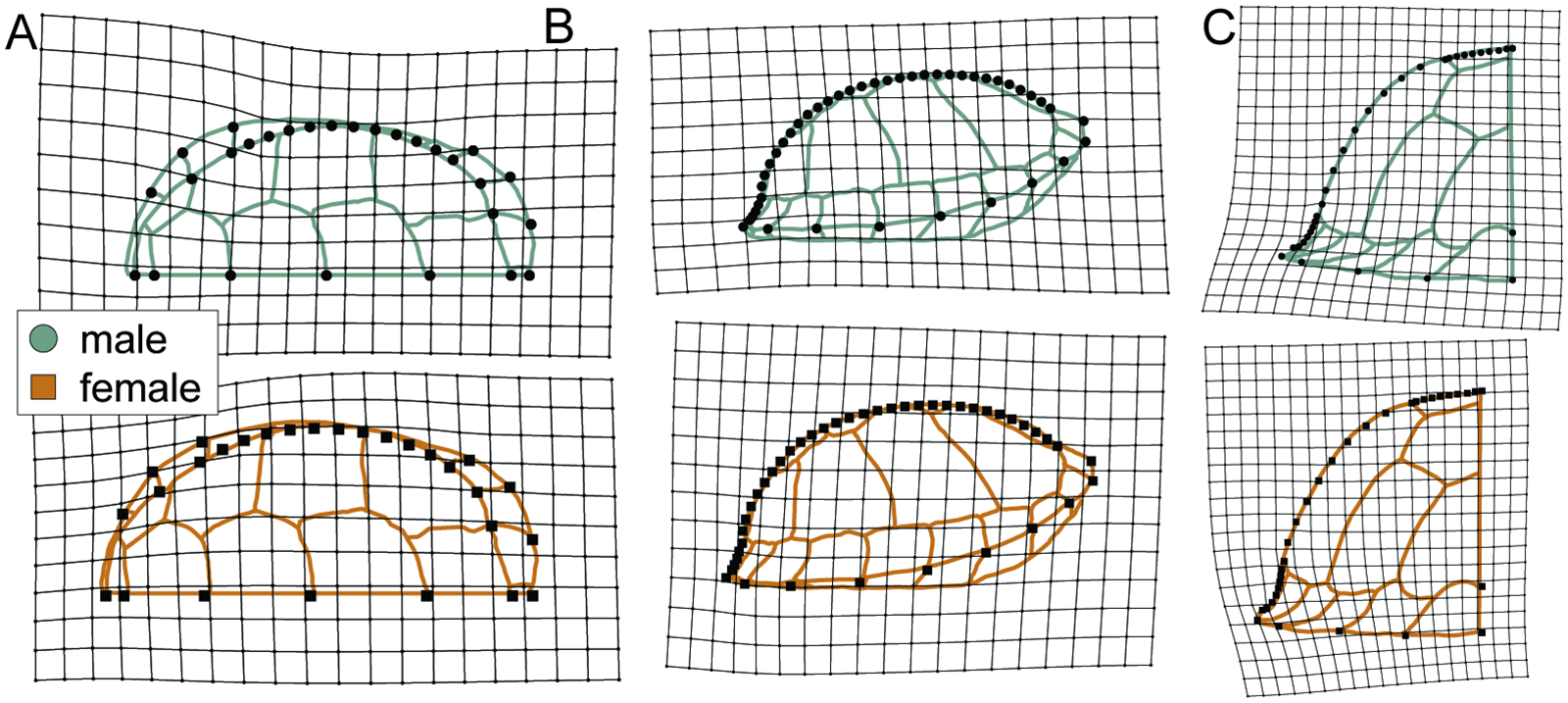
Mean shape differences due to sexual dimorphism in *T*. *carolina*. Line drawings indicate hypothetical carapaces consistent with each landmark configuration and are not exact predictions of most sulcus locations.

Seven of the fifty-two sexed specimens (13.5%) had plastron shapes inconsistent with their sex. Either males had flat plastra (5/7) or females had visible indentations in their plastra (2/7). Sexual size dimorphism was significant, with males slightly longer than females (p = 0.005) and a resulting SDI of -0.086 (Fig 4). After taking size and subspecies into account, sex had a significant effect on shape in all three views, accounting for 4–9% of variation (Table 4). There was no significant interaction between sex and subspecies identity or centroid size. Females have slightly taller shells and peripherals that do not project as far laterally as males but the two sexes have a similar anterior shell shape (Fig 5). Jackknife assignments tests that identified the sex of a specimen based on carapace shape had moderate levels of accuracy (dorsal view: 71.1% correct assignments, lateral and posterior views: 63.5% correct assignments; Table 5). Given the high amounts of error in assignment tests of sex and the low explanatory power of sex on shape, the two sexes were considered indistinguishable based on carapace shape alone. They were pooled in further analyses.

**Table 4:** Results of Procrustes ANOVA testing for differences between carapace shape at between sexes, with log-transformed centroid size and subspecies included as a covariates.

Factor	View	R^2^	F	p
Centroid Size	Dorsal	0.052	3.103	**0.033**
	Lateral	0.067	5.113	**0.024**
	Posterior	0.047	3.053	**0.03**
Subspecies	Dorsal	0.099	2.952	**0.009**
	Lateral	0.263	10.054	**0.001**
	Posterior	0.187	6.098	**0.001**
Sex	Dorsal	0.091	5.455	**0.003**
	Lateral	0.044	3.375	**0.011**
	Posterior	0.06	3.905	**0.001**
Centroid Size: Sex	Dorsal	0.008	0.488	0.701
	Lateral	0.03	2.317	0.069
	Posterior	0.016	1.063	0.278
Subspecies: Sex	Dorsal	0.013	0.402	0.911
	Lateral	0.022	0.828	0.465
	Posterior	0.016	0.532	0.873

P-values less than 0.05 are in bold. All p-values are corrected using the false discovery rate correction.

**Table 5:** Results of jackknife validated assignment tests of specimens to sex using CVA of carapace shape. Numbers in brackets are the percent of specimens assigned.

	View	Female	Male
Female	Dorsal	18 [69.2%]	8 [30.8%]
	Lateral	17 [65.4%]	9 [34.6%]
	Posterior	18 [69.2%]	8 [30.8%]
Male	Dorsal	7 [26.9%]	19 [73.1%]
	Lateral	10 [38.5%]	16 [61.5%]
	Posterior	11 [42.3%]	15 [57.7%]

### Modern geographic variation

**Fig 6:**
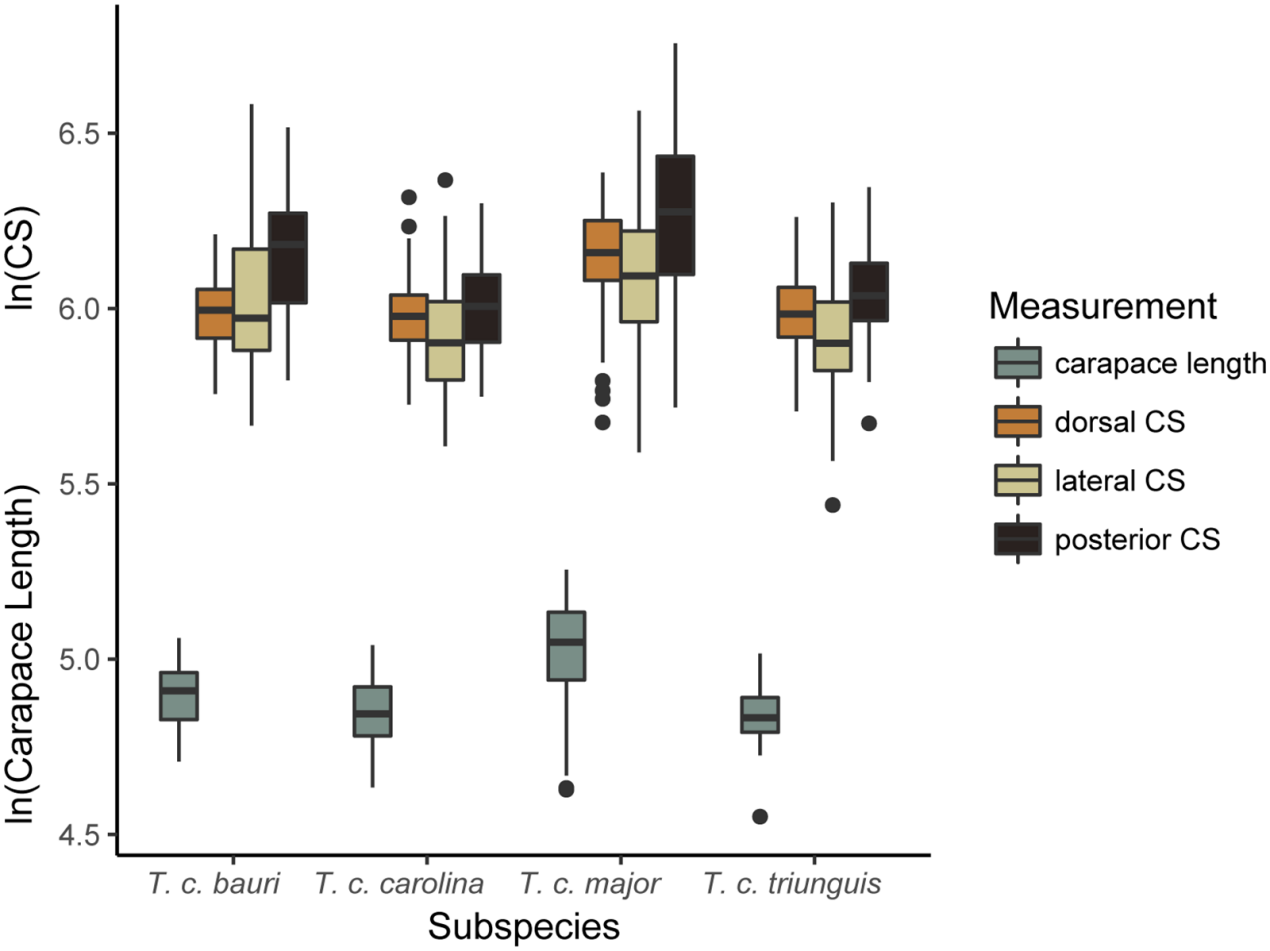
Box plots of four measures of carapace size of recognized species of *T*. *carolina* in the United States. CS = centroid size.

In terms of carapace length, ANOVA resulted in a significant difference in size between the four subspecies (Table 6). The subspecies with the longest carapace was *T*. *c*. *major*, followed by *T*. *c*. *bauri*, then *T*. *c*. *carolina* and *T*. *c*. *triunguis*, of which the mean lengths of the latter two differ by only one mm (*T*. *c*. *major* mean = 151 mm, range = 102–192 mm, *T*. *c*. *bauri* mean = 133 mm, range = 111–158 mm, *T*. *c*. *carolina* mean = 127 mm, range = 103–154 mm, *T*. *c*. *triunguis* mean = 126 mm, range = 95–160 mm). Box plots of multiple measures of carapace size (Fig 6) show that the range of sizes of *T*. *c*. *bauri*, *T*. *c*. *carolina*, and *T*. *c*. *triunguis* broadly overlap. Analyses of size performed using centroid size as a metric in three views of the carapace produced similar results (Table 6).

**Table 6:** Results of ANOVA testing for differences in various measures of carapace size between recognized subspecies of *T*. *carolina* in the United States.

Measurement	Df	F value	P
Carapace Length	3	26.37406	9.00E-14
Dorsal CS	3	17.52109	5.41E-10
Lateral CS	3	7.766471	6.31E-05
Posterior CS	3	21.72292	6.90E-12

CS = centroid size. All values were log-transformed prior to testing.

**Fig 7:**
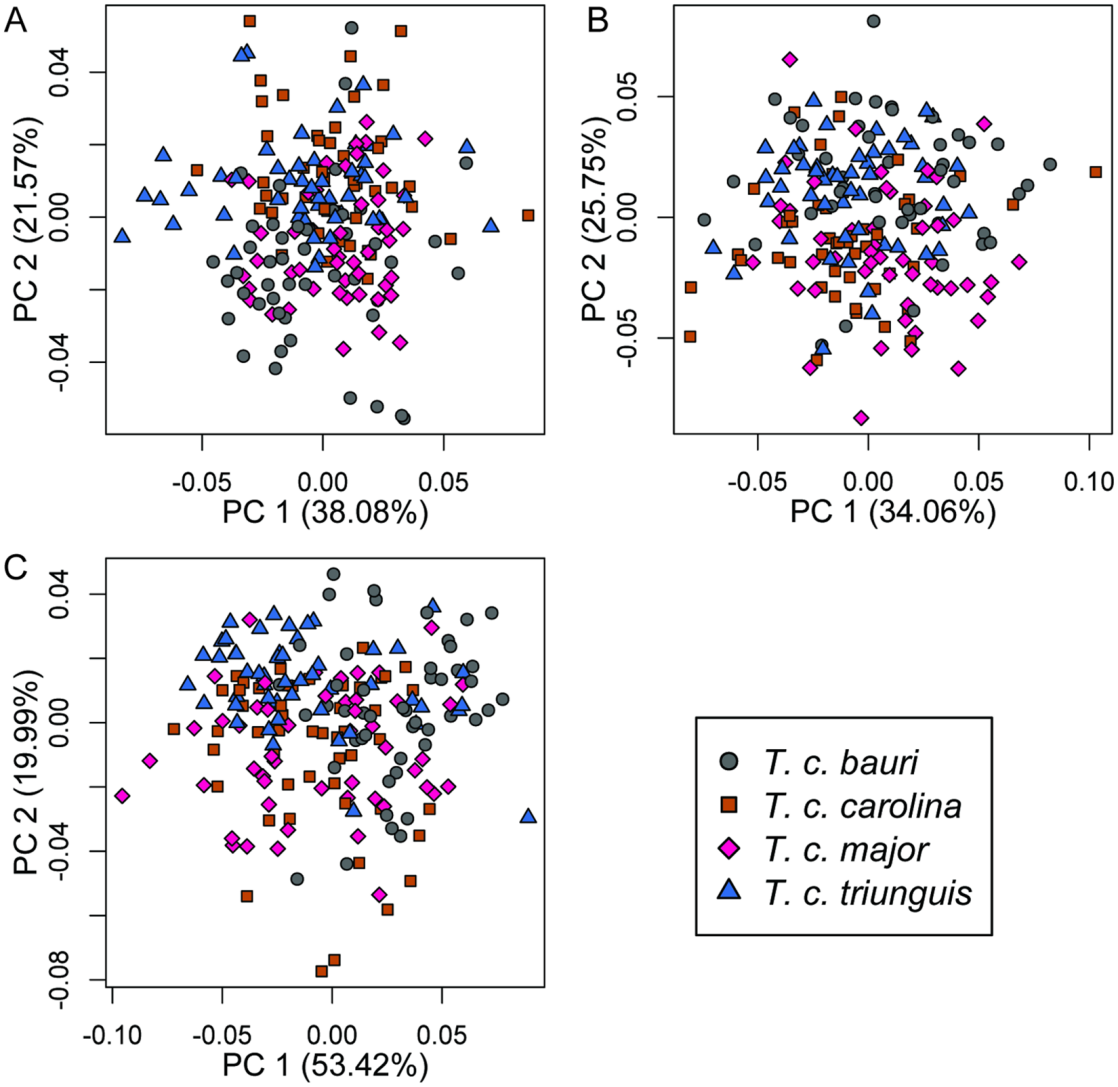
Principal components (PC) plots of variation in carapace shape between the four recognized species of *T*. *carolina* in the United States. (A) Dorsal view, (B) lateral view, and (C) posterior view of carapace.

In all three views, subspecies shape broadly overlapped in the first two PCs of morphospace (Fig 7). Jackknife validation of assignment tests resulted in low classification accuracy (57–59%, Table 7). The best clustering model recovered by *k*-means clustering contained only one group (BIC = 5380.3, 10504.4) in dorsal and lateral views. The best model including more than one cluster had lower BIC scores (ΔBIC = 21.5, 36.3, *k* = 2). In posterior view, a *k = 2* model had a better BIC score than the best *k* = 1 model (BIC = 18335.9, ΔBIC = 45.4), but the second of the two clusters in the *k* = 2 model consisted of only a single specimen. That model was rejected as uninformative and the next best model, *k* = 1, was preferred.

**Table 7:** Results of jackknife validated assignment test of specimens to subspecies using CVA of carapace shape.

Original Group	View	*T*. *c*. *bauri*	*T*. *c*. *carolina*	*T*. *c*. *major*	*T*. *c*. *triunguis*
*T*. *c*. *bauri*	Dorsal	65.3%	8.2%	24.5%	2.0%
	Lateral	59.2%	18.4%	10.2%	12.2%
	Posterior	61.2%	4.1%	18.4%	16.3%
*T*. *c*. *carolina*	Dorsal	10.0%	42.0%	24.0%	24.0%
	Lateral	16.0%	48.0%	24.0%	12.0%
	Posterior	8.0%	60.0%	20.0%	12.0%
*T*. *c*. *major*	Dorsal	20.0%	14.0%	56.0%	10.0%
	Lateral	12.0%	20.0%	58.0%	10.0%
	Posterior	28.0%	18.0%	40.0%	14.0%
*T*. *c*. *triunguis*	Dorsal	2.0%	21.6%	11.8%	64.7%
	Lateral	6.1%	4.1%	16.3%	73.5%
	Posterior	19.6%	5.9%	11.8%	62.7%

**Fig 8:**
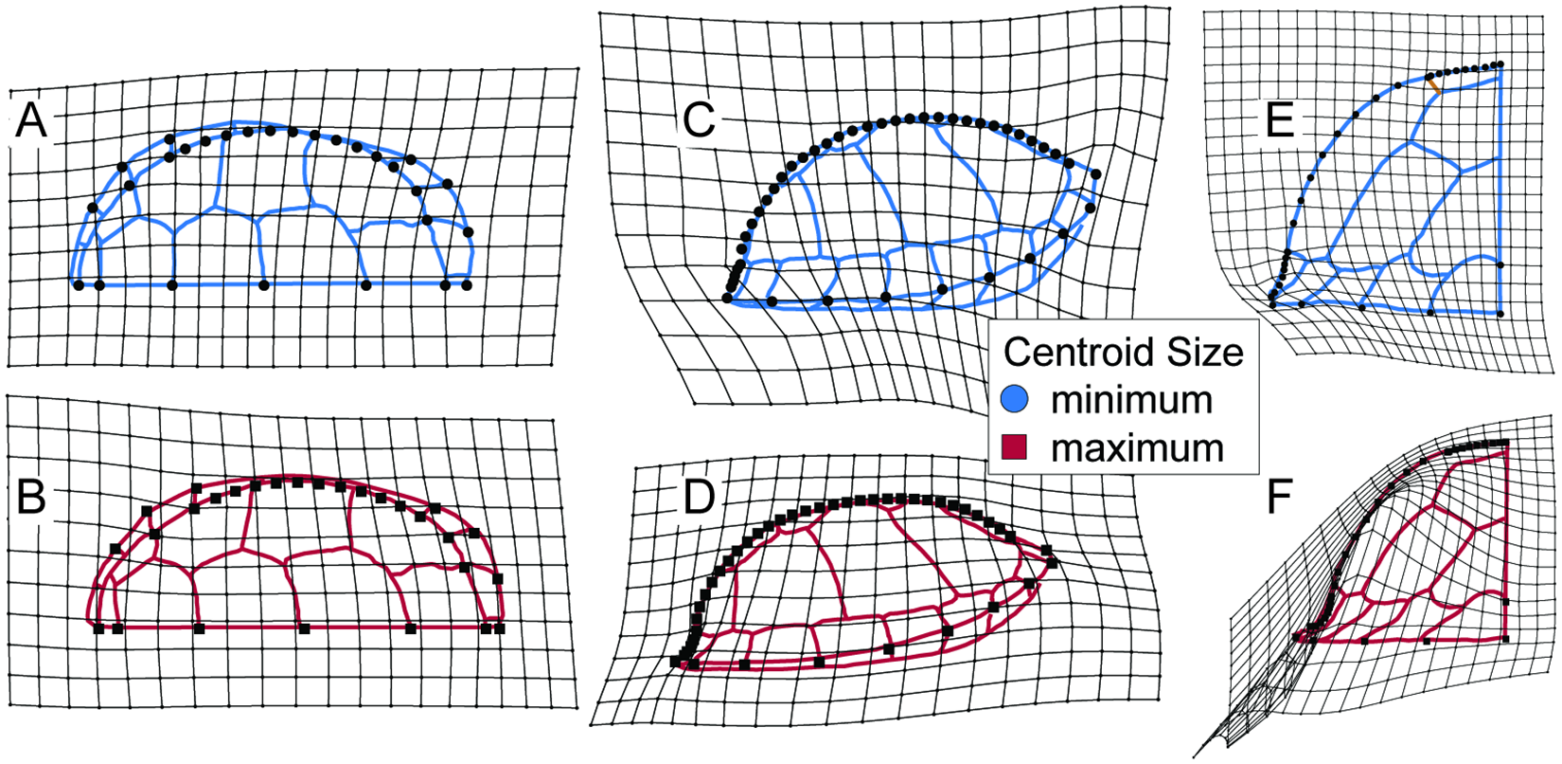
Predicted shape differences due to allometry in carapaces of modern *T*. *carolina*. Thin plate splines show deformation between minimum and maximum centroid sizes relative to each other in (A, B) dorsal, (C, D) lateral, and (E, F) posterior views. Line drawings indicate hypothetical carapaces consistent with each landmark configuration and are not exact predictions of all sulcus locations.

Allometric variation was significant in all three views, explaining 3–8% of total shape variation. At smaller sizes, the allometric model predicted a relatively anteroposteriorly shorter, dorsoventrally taller, and mediolaterally wider shell. The model also predicted carapaces with less externally curved, or concave, peripherals, a feature referred to as “flaring” [29] (Fig 8). The allometric model predicted the presence of a midline dorsal keel at small sizes and the loss of a midline dorsal keel at the largest sizes.

The two most important SEs together explained 7–10% of shape variation in each view (Table 8). Forward selection of spatial eigenvectors to explain variation in shape chose SE2 and SE3 in dorsal view and SE1 and SE2 in lateral and posterior views. Procrustes ANOVA that did not include a spatial covariate recovered variably significant differences between some subspecies pairs (Table 9). Adding a single SE as a covariate (dorsal = SE3, lateral and posterior = SE2) rendered any pairwise differences between subspecies nonsignificant (Table 9).

**Table 8:** Results of Procrustes ANOVA testing for significance of continuous covariates of carapace shape.

Factor		View	R^2^	F	p
First SE	SE2	Dorsal	0.047	10.248	**0.001**
	SE1	Lateral	0.038	8.81	**0.001**
	SE1	Posterior	0.047	10.704	**0.001**
Second SE	SE3	Dorsal	0.03	6.412	**0.001**
	SE2	Lateral	0.038	8.76	**0.001**
	SE2	Posterior	0.061	13.942	**0.001**
Centroid Size		Dorsal	0.022	4.746	**0.003**
		Lateral	0.079	18.281	**0.001**
		Posterior	0.032	7.26	**0.003**

SE = Spatial Eigenvectors. SEs are the most important for explaining shape variance and were chosen for each view based on forward selection. They are reported in order of importance for each view. P-values less than 0.05 are in bold.

**Table 9:** P-values of pairwise comparisons of shape between subspecies of *T*. *carolina* using Procrustes ANOVA. Lower triangle shows results of analysis with no spatial eigenvector covariate included. Upper triangle shows results of analysis with spatial eigenvector covariate included.

Original Group	View	*T*. *c*. *bauri*	*T*. *c*. *carolina*	*T*. *c*. *major*	*T*. *c*. *triunguis*
*T*. *c*. *bauri*	Dorsal	-	1	1	1
	Lateral	-	0.368	1	1
	Posterior	-	0.59	0.779	1
*T*. *c*. *carolina*	Dorsal	0.318	-	1	1
	Lateral	**0.016**	-	0.856	0.792
	Posterior	0.964	-	0.779	0.992
*T*. *c*. *major*	Dorsal	0.308	0.828	-	1
	Lateral	0.179	0.452	-	1
	Posterior	0.964	0.929	-	0.992
*T*. *c*. *triunguis*	Dorsal	0.781	0.184	0.088	-
	Lateral	**0.004**	0.831	0.268	-
	Posterior	0.964	0.929	1	-

P-values adjusted using the false discovery rate correction. P-values < 0.05 indicated in bold.

**Fig 9:**
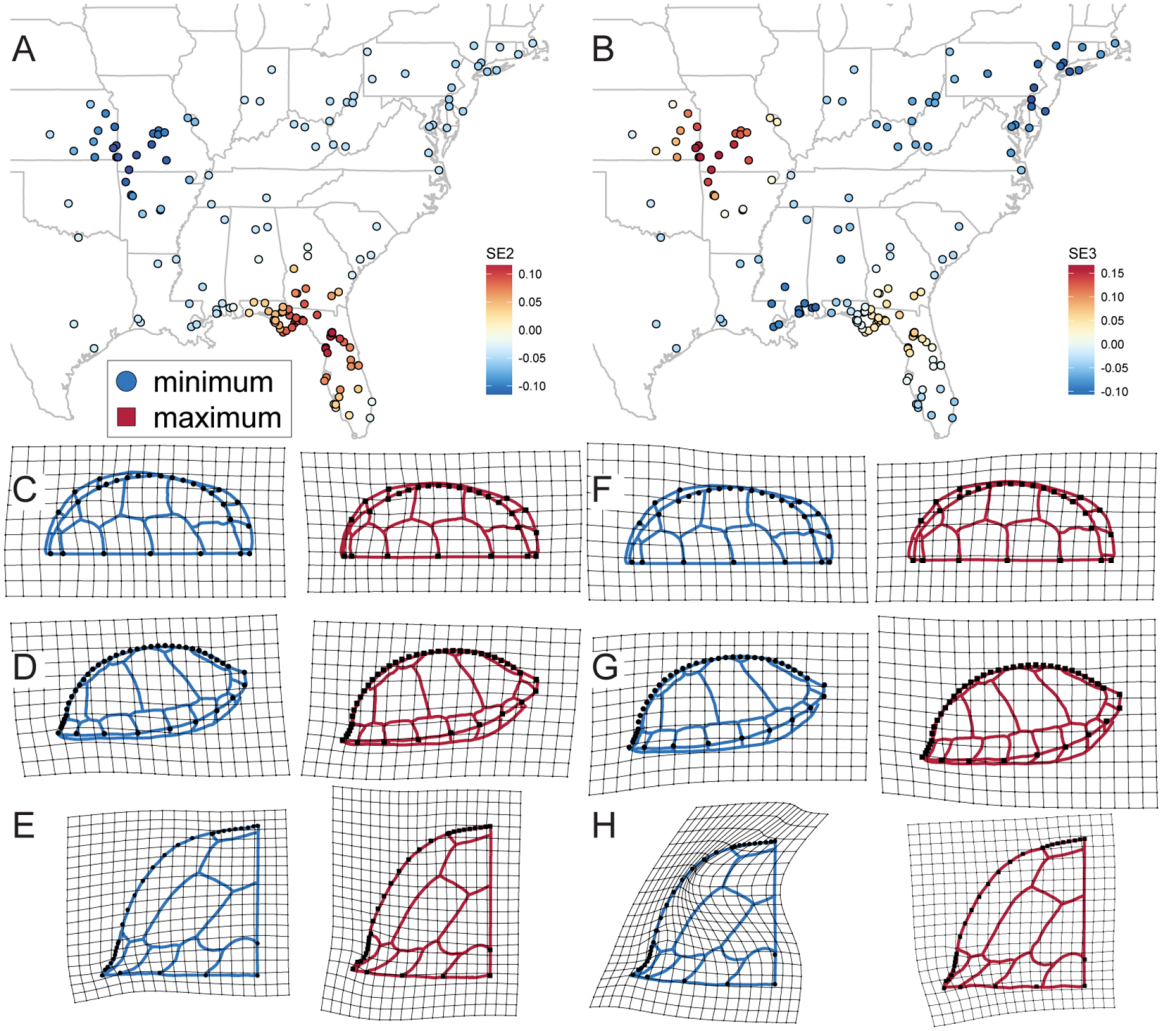
Geographic representation of the two spatial eigenvectors (SEs) that account for subspecific differences in shape and shape differences predicted by those SEs in carapaces of modern *T*. *carolina*. Predicted mean shapes show maximum and minimum values of (A) SE2 and (B) SE3 in (C, F) dorsal, (D, G) lateral, and (E, H) posterior views, respectively. Line drawings indicate hypothetical carapaces consistent with each landmark configuration and are not exact predictions of all sulcus locations.

SE1 demarcated two sampling clusters, one in peninsular Florida and one in the Florida panhandle [22]. The rest of the species range of *T*. *carolina* had SE1 values near zero. SE2 described a spatial gradient between a region around southwestern Arkansas and a region in north-central Florida (Fig 9). Differences between the two end-member shapes predicted by SE2 were concentrated in the degree of peripheral flaring and relative carapace width. SE3 described a gradient between three regions. At one extreme was the same region around southwestern Arkansas described by SE2. The other extreme of the gradient was located both around the mouth of the Mississippi River and in the northeastern United States in a region centered on New Jersey (Fig 9). The two end member carapace shapes predicted by SE3 differed in the degree to which the peripherals angle outward or are not confluent with the costals, a feature also referred to as “flaring” [22]. The two shapes also differed in shell height and the degree to which the posterior profile of the shell is either gently sloping or is “boxier” with a more squared off or sharply sloping profile (Fig 9). Shape differences predicted by SE3 follow a similar pattern to shape changes associated with allometry (Fig 8).

### Variation in the fossil record

**Fig 10:**
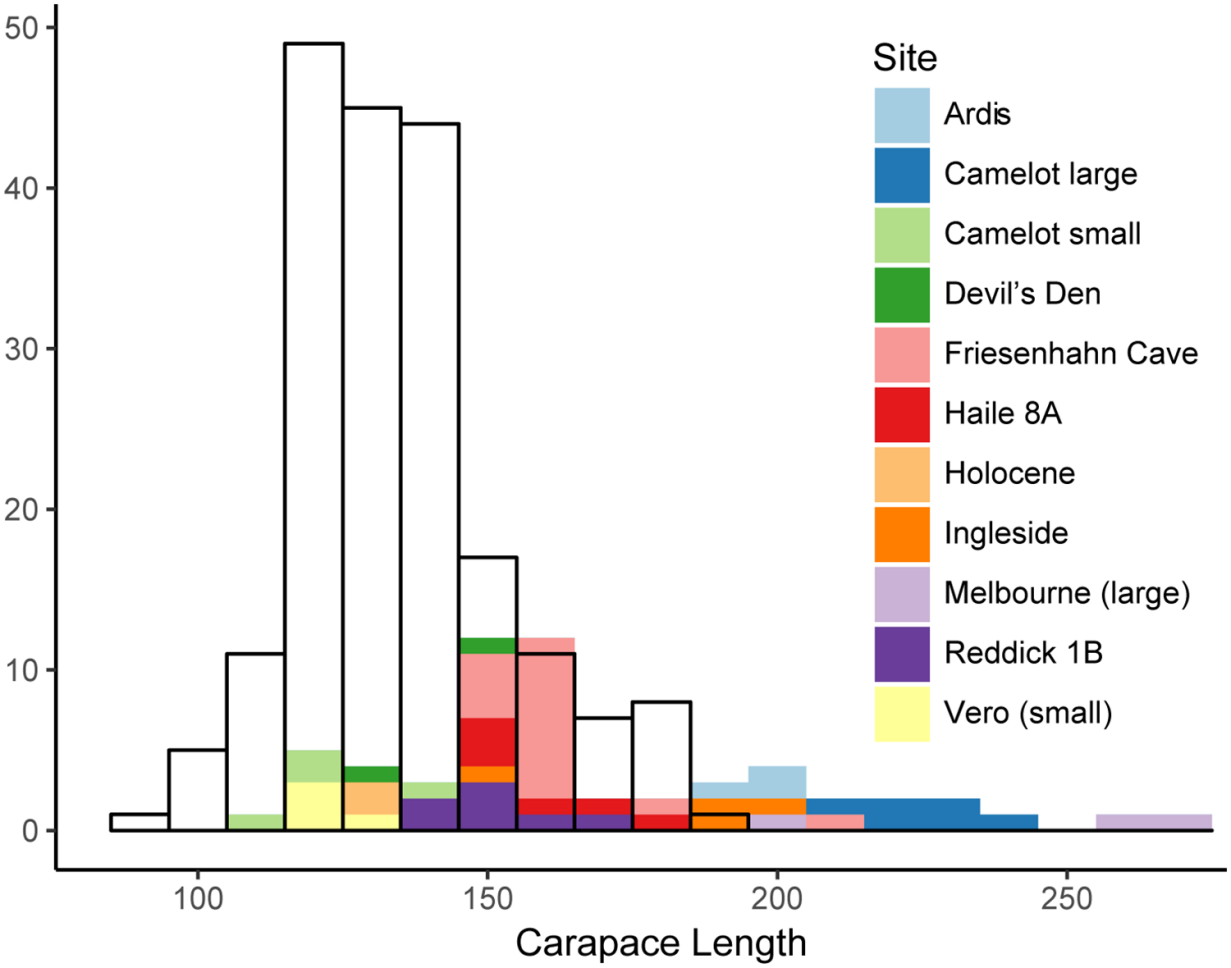
Histogram of carapace lengths of modern and fossil *T*. *carolina*. Transparent bars show distribution of sampled modern specimens. Colored bars show distribution of specimens from sampled fossil localities.

Fossils with closed carapaces occupied a wider range of carapace lengths than the comparable sample of modern specimens (Fig 10, modern carapace length 94.7–191.5 mm, fossil carapace length 112.9–270.2 mm). In two cases, the Vero-Melbourne site pair and the Camelot site, carapace length distribution was highly bimodal. The distributions centered on the two means did not overlap (Fig 10). When the bimodality was modelled as sexual size dimorphism, index values were -0.960 and -0.818, respectively (Fig 4). Those SDIs were outside the resampled confidence intervals of SDIs of modern specimens (Fig 4), and therefore sexual dimorphism could not account for the bimodality of sizes at the site. Given the shell closure in all specimens and lack of intermediate-sized individuals, the bimodality could also not be explained by variation between juveniles and adults. Large and small morphs at those sites were treated separately in further analyses.

**Fig 11:**
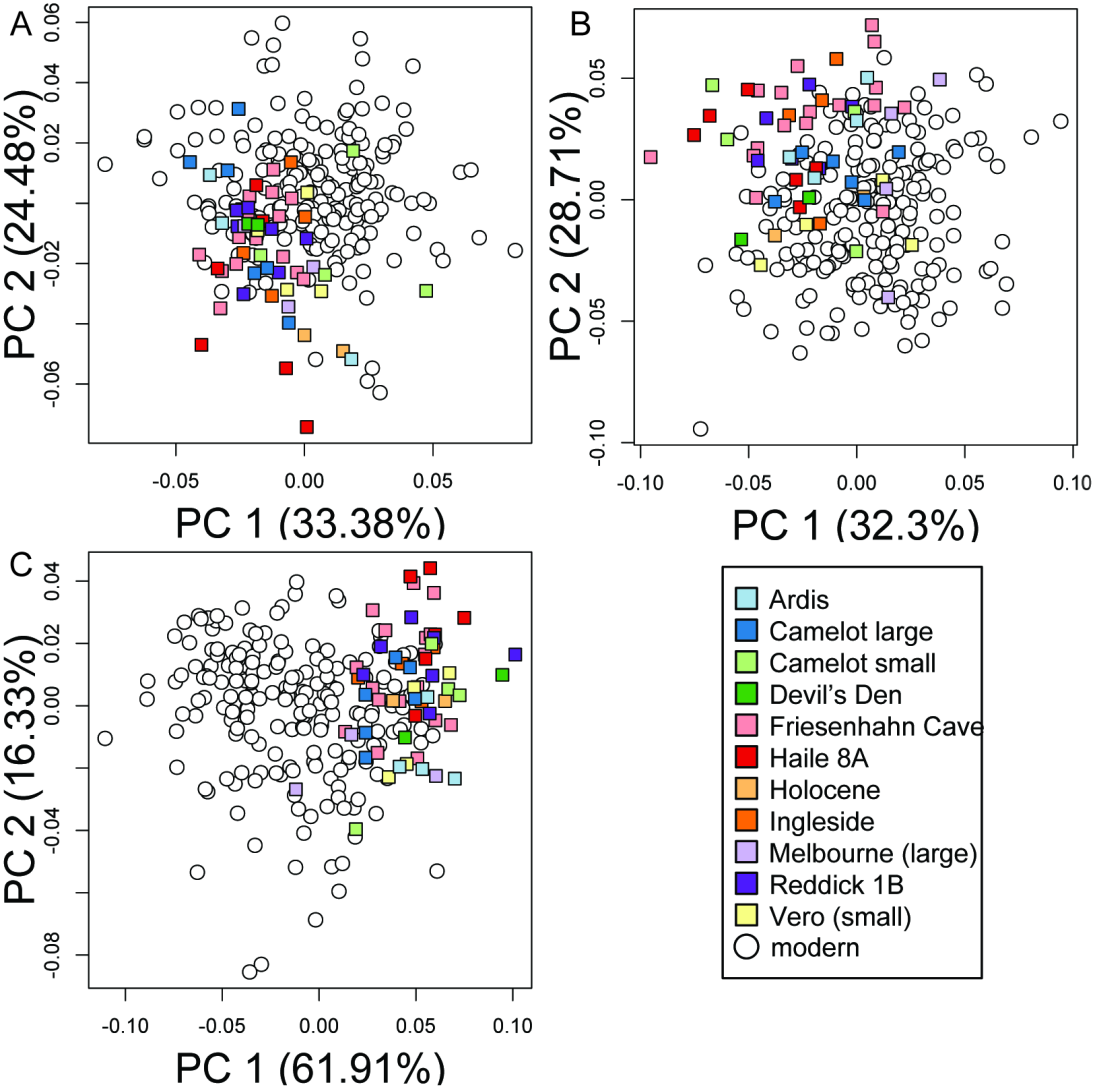
Principal components (PC) plots of variation in carapace shape in modern and fossil *T*. *carolina*. (A) Dorsal view, (B) lateral view, and (C) posterior view of carapace.

Comparisons of slopes resulted in significant differences between size-shape relationships in the modern and fossilized samples in two of three views (dorsal: p = 0.022, lateral p = 0.037, posterior p = 0.139). Subsequently, the component of shape variation predicted by the growth trajectory of the modern sample was removed from all specimens and those allometrically corrected shapes were used in downstream analyses. The fossil sample overlapped with a subset of the modern sample in the first two PCs (Fig 11). *K*-means cluster analyses resulted in *k* = 1 group models outperforming multi-group models for each view (in dorsal, lateral, and posterior view, respectively: BIC = 5380.3, 3100.2, 5469.3, ΔBIC = 21.5, 17.8, 2.5).

When allometrically corrected shapes of specimens from each fossil site or morph within site were compared to allometrically corrected modern specimens using pairwise Procrustes ANOVA, only Holocene specimens were not significantly different from modern specimens in at least one view (Table 10). Specimens from Friesenhahn Cave, Haile 8A, and Reddick 1B were consistently significantly different from modern specimens. Those three sites also significantly differed from specimens from Melbourne in lateral view. No other pairwise differences between fossil sites were significant.

**Table 10:** P-values of pairwise comparisons of shape between Pleistocene fossils of *T*. *carolina* from different sites using Procrustes ANOVA.

Site	N	Ardis (N = 4)	Camelot (large)	Camelot (small)	Devil’s Den	Friesenhahn Cave	Haile 8A	Holocene	Ingleside	Melbourne (large	Reddick 1B	Vero (small)
Camelot (large)	6	0.984	-									
		0.853	-									
		0.797	-									
Camelot (small)	4	0.396	0.136	-								
		0.695	0.464	-								
		0.878	0.708	-								
Devil’s Den	2	0.984	1	0.333	-							
		0.602	0.464	0.635	-							
		0.878	0.708	1	-							
Friesenhahn Cave	17	0.458	0.17	0.132	0.696	-						
		0.695	0.204	0.597	0.276	-						
		0.797	0.708	1	0.89	-						
Haile 8A	6	0.566	0.17	0.132	0.696	0.473	-					
		0.602	0.204	0.793	0.38	0.212	-					
		0.797	0.708	1	0.89	0.877	-					
Holocene	2	0.458	0.17	0.384	0.696	0.276	0.472	-				
		0.695	0.6	0.635	0.636	0.308	0.464	-				
		0.878	0.708	1	0.89	0.937	0.926	-				
Ingleside	4	0.566	0.396	0.384	0.829	0.808	0.472	0.425	-			
		0.983	0.464	0.635	0.366	0.995	0.464	0.727	-			
		0.804	0.986	1	0.89	0.877	0.926	1	-			
Melbourne (large)	4	0.567	0.267	0.328	0.696	0.177	0.472	0.496	0.609	-		
		0.602	0.464	0.084	0.276	**0.048**	**0.018**	0.727	0.474	-		
		0.878	0.708	1	0.89	0.877	0.926	1	1	-		
Reddick 1B	6	0.458	0.199	0.23	0.696	0.818	0.502	0.393	0.758	0.356	-	
		0.695	0.261	0.793	0.276	0.238	0.704	0.727	0.648	**0.032**	-	
		0.797	0.742	1	0.89	0.877	1	1	1	0.776	-	
Vero (small)	4	0.44	0.136	0.384	0.696	0.177	0.351	0.393	0.609	0.375	0.377	-
		0.602	0.464	0.404	0.38	0.068	0.171	0.976	0.474	0.204	0.209	-
		0.878	0.708	1	0.89	0.877	0.926	1	1	0.776	1	-
Modern		0.052	**0.012**	0.384	0.3	**0.003**	**0.003**	0.079	0.123	0.052	**0.003**	0.105
		**0.038**	0.204	**0.029**	0.12	**0.004**	**0.004**	0.661	**0.029**	**0.045**	**0.004**	0.379
		**0.002**	**0.012**	**0.005**	**0.008**	**0.002**	**0.002**	0.05	**0.011**	0.058	**0.002**	**0.004**

Each row within a site presents values for dorsal, lateral, and posterior view in that order. P-values adjusted using the false discovery rate correction. P-values < 0.05 indicated in bold.

**Fig 12:**
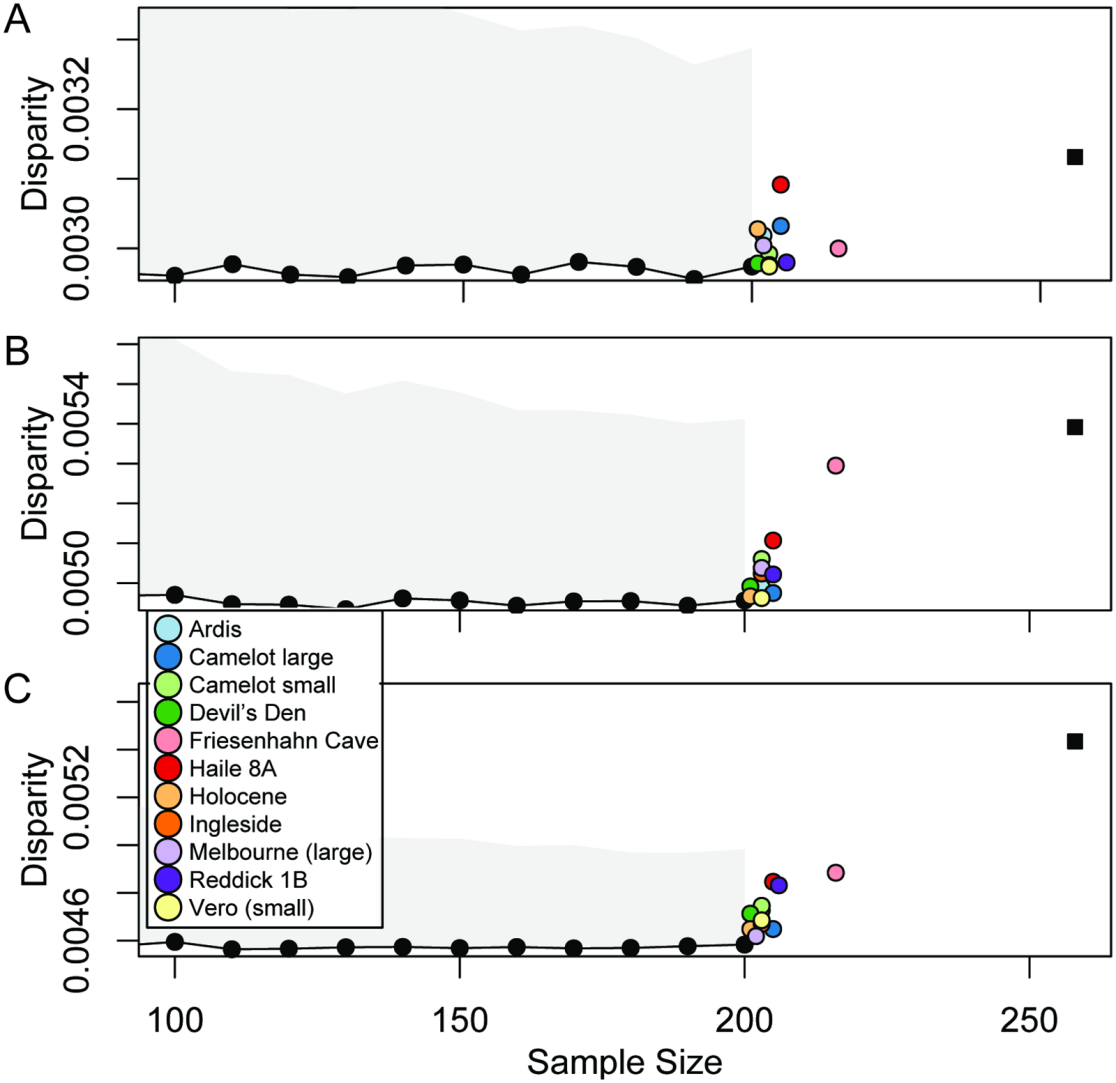
Total disparity of datasets including 200 modern carapaces of *T*. *carolina* and a given sample of carapaces from various fossil sites. Black circles connected by lines show mean estimates of disparity calculated from resampling the modern dataset at a sample size indicated by the x-axis. Shaded area indicates a one-tailed 95% confidence interval also derived from resampling the modern dataset. Colored circles indicate disparity values resulting from the addition of specimens from a given fossil site or morph to the full, modern dataset. Black square indicates disparity resulting from the addition of all 57 fossils to the modern datasets in (A) dorsal, (B) lateral, and (C) posterior views.

Total shape disparity of extant specimens was stable regardless of sample size (Fig 12), although the confidence interval became narrower with increasing sample size. No individual fossil site increased total disparity beyond the 95% confidence interval when added to the sample, but all fossils added together did so in posterior view and potentially in lateral view. Some sites, notably Friesenhahn Cave and Haile 8A, consistently increased total disparity when added to the dataset though not enough to be statistically significant.

Haile 8A was chosen as a representative of the novel morphology in the fossil record because it consistently occupied novel portions of the first and second PCs in comparison modern specimens (Fig 11). It was also significantly different from modern specimens in all three views (Table 10) and it consistently increased the amount of morphospace occupied when added to the dataset of modern specimens (Fig 12). In comparison to mean modern specimen shape, specimens from Haile 8A are anteroposteriorly shorter, dorsoventrally taller, and mediolaterally wider with peripherals that are slightly concave but are confluent with the downward curve of the costals. The relative increase in the height of the shell is greater than the relative increase in width. That difference makes the shell appear narrower than the mean modern specimen in posterior view.

## Discussion

### Shell closure a proxy for reproductive maturity

Studies of modern and fossilized *T*. *carolina* use different approaches to separating reproductively immature and mature individuals, or juveniles and adults [157]. That use of divergent methods impedes comparison of results between different studies. In the fossil record, evidence for the juvenile nature of a specimen is unstated or based on the degree of fusion of the shell [29,30,158]. In other contexts, closure of carapace fontanelles has been suggested as another proxy of sexual maturity in turtles, but has not been widely applied, at least explicitly, to fossils [85]. In modern populations carapace length is one commonly used proxy for reproductive maturity [85,159–161]. It is complemented by counts of growth rings, which act as a proxy of age and another correlate of sexual maturity [85,162].

All three proxies have potential problems in their use across different records. Length at sexual maturity varies from population to population [82,159–161], making it impossible to use a single value to accurately identify reproductively mature individuals across multiple populations through both space and time. Accuracy of growth ring counts as proxies for age declines after turtles reach reproductive maturity and growth rings are rarely preserved in the fossil record [162,163]. The evidence for use of skeletal changes as a proxy is sparse [85]. With further tests of its utility, skeletal changes provide a promising proxy to use across a range of spatial and temporal samples.

In studies of the modern biota, previous divisions between reproductively immature and mature individuals were set between 97 and 120 mm carapace lengths [82,159–161,164], which corresponded to 5–8 MGR [84,160,161]. Previous researchers also reported that after the attainment of 9–15 MGR, soon after the attainment of reproductive maturity, the relationship between number of MGR and size become less tightly correlated [84,161,163]. The range of observations is consistent between specimens from Florida, Missouri, Maryland, North Carolina, and New York, corresponding to the recognized subspecies *T*. *c*. *bauri*, *T*. *c*. *triunguis*, and *T*. *c*. *carolina* [84,159–161,163,164]. Results presented here are also consistent with those reports. In the species-wide sample used in this study, the attainment of 8 MGR and a carapace length of at least 100 cm on average marks a change in relationship between these two proxies in this study. After this division any potential relationship between MGR and carapace length loses statistical significance. That change is also marked by closure of carapacial fontanelles. I do not find strong support for the use of fusion, as opposed to fontanelle closure, as a reliable proxy for number of MGR, shell length, or by extension, age or attainment of sexual maturity. The concordant changes in relationships between all three proxies when the carapace closes supports the hypothesis that this skeletal trait should be added as a proxy for maturity in *T*. *carolina* across spatiotemporal settings. Carapacial closure is a particularly attractive feature because it can be measured in both fossilized and modern osteological specimens.

One limitation of the results presented here is the comparison of morphological features across individuals without direct knowledge of the age or reproductive status of the individuals. Previous research about the utility of MGR and size are used as a baseline against which to test the correspondence of shell closure to those patterns. Ideally, future research would track changes in shell closure with multiple measures of individuals over time.

### Sexual dimorphism weak in *T*. *carolina*

In the modern biota, male and female *T*. *carolina* are differentiated by iris and head coloration, tail and hind claw dimensions, size, and plastron indentation [24,40,84,163]. Only one of these features, plastron indentation, is practically applicable to the fossil record. Plastron indentation is commonly used to determine the sex of modern individuals (e.g., [141,165]). Both regional and intra-population variation is present in the reliability of this feature, but the relative number of individuals with problematic plastron morphology given their sex is reported to be small [40,84,163].

Consistent with previous results, some error occurred when plastron indentation was used as the sole indicator of an individual’s sex. The error rate was relatively low (13.5%) for individuals in the southern and eastern portion of the species’ range (*T*. *c*. *carolina*, *T*. *c*. *bauri*, *T*. *c*. *major*). However, that rate may be artificially low and may not be an accurate indicator of reliability across the entire geographic range of the species. First, accuracy was judged based on museum catalog records instead of direct observation of reproductive organs. It is possible that catalogue errors could have spuriously contributed to the error rate. Such circularity is more likely to make observations of sex-inconsistent plastral morphology less frequent. Second, males from the northwestern part of the species’ range (*T*. *c*. *triunguis*) are reported to have little or no plastral indentation [22]. *Terrapene carolina triunguis* was the only subspecies with not enough specimens identified to sex to include in the study of sexual dimorphism. Given the error present in the three measured subspecies and reports of more instances of sex-inconsistent plastron morphology in *T*. *c*. *triunguis*, the use of plastron morphology to sex individuals in the absence of other secondary sexual characteristics, such as is the case in the fossil record, should be used with caution.

In this study, modern *T*. *carolina* displayed generally weak male-biased sexual size dimorphism, consistent with most previous results [45,141,160,161,166–171]. The recalculated SDI of -0.13 from a previous study [141] is within the confidence interval of results presented here (Fig 4). Reported mean carapace lengths from a study in which females were larger than males correspond to an SDI of 0.11 [172], a figure also within the confidence interval modelled in this study (Fig 4). Growth rates and age at maturity are subject to ecophenotypic plasticity [170], which may increase the variation in observed SDI in different populations.

In some previous reports of sexual shape dimorphism, males have significantly dorsoventrally lower, mediolaterally narrower carapaces with more strongly flared peripherals at a given size [45,160,161,168]. In other studies, no significant shape differences between the sexes was reported [32,169,171]. Across populations, sexual dimorphism is statistically significant in this study. Males had, on average, slightly dorsoventrally lower carapaces with slightly more laterally extending, or flared, peripherals (Fig 5). Peripherals aside, the average male carapace was not narrower than the average female carapace (Fig 5A). Furthermore, the range of variation around these mean shapes were wide, resulting in low assignment accuracy. In that context, the difference between males and females is not distinct enough to allow for unambiguous identification to sex in the fossil record of *T*. *carolina* based on carapace shape alone.

### Subspecies carapaces are not diagnostic

Although a statistically significant amount of shape variation was explained by the presence of currently recognized modern subspecies in an aspatial framework for both the sexual dimorphism (N = 60) and subspecies dataset (N = 200), additional evidence supports the hypothesis that the subspecies system is not the best way to study geographical variation in carapace shape of *T*. *carolina*. First, incorporation of spatial autocorrelation into the Procrustes ANOVA that initially supported significant differences between subspecies rendered those differences insignificant (Table 9). Second, additional analyses do not corroborate the hypothesis that there are diagnosable differences between the shapes of carapaces of the four subspecies. Subspecies broadly overlapped in the first two principal components in all views. Model-based clustering analyses recovered a single best group containing all specimens. Classification accuracy of subspecies identification based on canonical variates was poor [173]. Only a little over half of the assignments of specimens to subspecies were correct (Table 5).

These results are not an evaluation of the existence of the subspecies of *T*. *carolina*. Their recognition in the modern biota is supported by other lines of evidence, primarily genetic data soft-tissue data [32,33], which are beyond the scope of this study. Instead, the evidence in this study does not support the recognition of the four subspecies in the fossil record based on carapace morphology, contrary to previous evaluations [22,29].

Instead of distinct subspecies, primary patterns of spatial variation in carapace shape are more consistent with spatially autocorrelated, clinal variation (Fig 9) [13,52,174,175]. General explanations for spatial autocorrelation are underlying patterns in neutral gene flow or local environmental conditions, both of which are geographically mediated [150]. In theory an SE could describe subspecies ranges, but in this study the two SEs that best account for subspecific variation in carapace shape do not describe the patterns of genetic variation that delimit subspecies [32,33]. Instead, the two principal clines describe differences between the northwestern extreme of the species range, which partially corresponds to *T*. *c*. *triunguis*, and combinations of parts of the Gulf Coast ranges of *T*. *c*. *major* and *T*. *c*. *bauri* (SE2, SE3) and the northeastern extreme of the species range, which partially corresponds to *T*. *c*. *carolina* (Fig 9) [32]. Furthermore, the turtles of the Gulf Coast at one extreme of each SE, putatively *T*. *c*. *major*, form an admixture of both genotypes and phenotypes from the other three subspecies as opposed to a diagnosable, well-supported subspecies [32,33]. It may be that local environmental conditions may better describe the geographic variation observed here, but those analyses are beyond the scope of this study.

The patterns of shape variation predicted by the geographic variation contained in SEs 2 and 3 are consistent with some previously published subspecies diagnoses but conflict with others [22,29,32]. Given that the different published diagnoses conflict with each other in some regards, it would be impossible to conform to all diagnoses (Table 1). The relatively mediolaterally narrower, dorsoventrally taller carapace associated with high values of SE2 (Fig 9) is consistent with results of significantly greater carapace depth previously ascribed to *T*. *c*. *bauri* (Table 1) [32]. The carapace of *T*. *c*. *triunguis* is also described as narrow, but both SEs predict the opposite pattern. The proposed elongate carapace with flared peripherals of *T*. *c*. *major* is also described by more negative values of SE3, but those same character could also be described by patterns of allometry [32].

Size plays a significant role in explaining shape variation. It continues to explain a significant component of shape variation even when accounting for spatial autocorrelation (Table 8). In particular, the allometric model recovered here is consistent with the previously proposed hypothesis that the putative subspecies *T*. *c*. *major* describes large turtles rather than genetically discrete turtles [32]. Allometric patterns can account for the elongate, flattened carapace with flared peripherals proposed for *T*. *c*. *major* (Table 1, Fig 8). Other researchers of allometry in emydid turtles found results similar to those reported above. Size explained much of the variation in each of three species of aquatic turtles [36]. It was also noted that species tended to become boxier with increasing age and that larger individuals were more elongate than smaller ones [36], as is documented here. The ‘filling in’ of an imaginary box around the shell can be seen most clearly in the visualization of the posterior view of *T*. *carolina* (Fig 8). The strength of the pattern and its similarity with allometric patterns in other emydid turtles support the hypothesis that allometric variation is a fundamental component of variation in *T*. *carolina* and can be applied to its fossil record.

### Novel morphology in the fossil record

Some of the results of this study are consistent with the interpretation of fossilized *T*. *carolina* as reflective of modern, standing diversity [24,30]. The Holocene specimens from peninsular Florida are not significantly different from modern specimens. Those results might be expected given their young geologic age. Other relatively geologically young specimens from Devil’s Den, Vero, and Melbourne are significantly different from modern specimen in one view but not the other two. Although most specimens from Melbourne are larger than any modern specimen included in this study, when carapace shape was corrected for allometric growth the fossils entirely overlap with modern specimens in the first two PCs (Fig 11). Specimens from Melbourne were notably similar to shapes predicted by patterns of allometric growth in modern specimens in terms of a dorsoventrally low, anteroposteriorly elongated shell with strongly concave, curved peripherals (compare Fig 8 to [42]).

Other morphology in the fossil record was not predicted by patterns of modern variation. The referral of large fossilized specimens to *T*. *c*. *major* [30] implies that the shape of those individuals should be predictable from an allometric growth trajectory shared with modern *T*. *carolina*. Those fossils that are larger than modern specimens should match a simple extension of that shared trajectory. The allometric slope predicted by large fossilized turtles is significantly different from the allometric growth trajectory of modern turtles. Therefore, large size in the fossil record is not solely the product of more growth. Growth can potentially account for shape differences in some large fossils, such as those at Melbourne, but not for most others.

**Fig 13:**
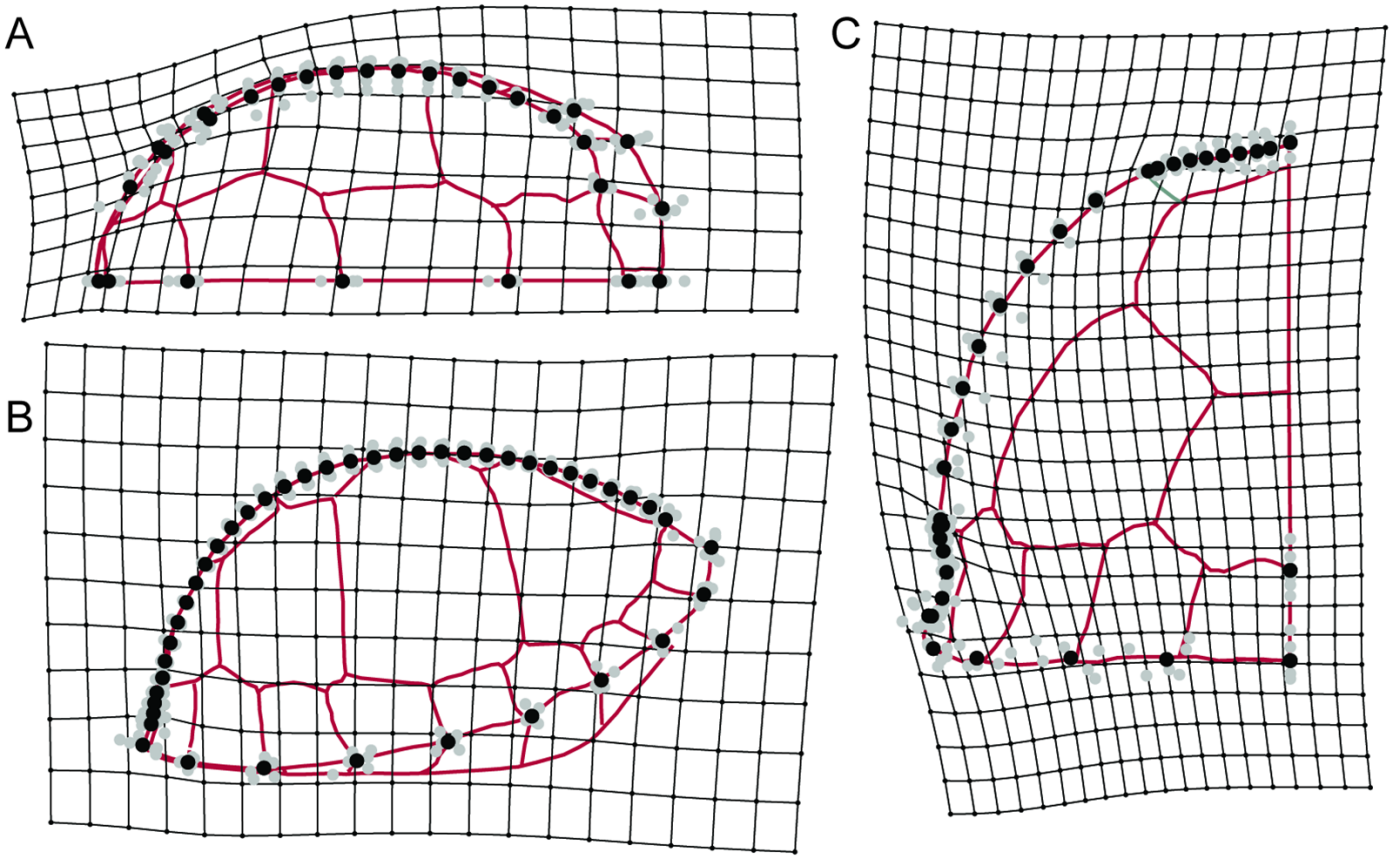
Comparison of mean carapace shape of modern *T*. *carolina* and fossils from Haile 8A. Thin plate splines show deformation from mean modern shape to mean shape at Haile 8A. Gray points show shapes of individual specimens from Haile 8A to indicate variation around the mean shape in (A) dorsal, (B) lateral, and (C) posterior view. Line drawings based on UF 3136.

This difference is illustrated by fossilized specimens from Haile 8A (Fig 13). The fossils are dorsoventrally taller and anteroposteriorly shorter than modern specimens. They also have prominent mid-dorsal keels and peripherals that meet one definition of flaring (concave and curled) but not another (angled away from the costals). Specimens from Haile 8A, Reddick 1B, and Friesenhahn Cave are larger on average than modern *T*. *carolina* (Fig 10), but when size is removed as a component of shape through both Procrustes superposition and allometric modelling, the remaining shapes are still different (Table 10, Fig 11). In addition, several of these specimens are within the size range observed in the modern biota. Therefore, the differences are unlikely to be due to extrapolation error.

The shape of those large specimens is not also described by either of the illustrated SEs (Fig 9). It is possible that the shape can be accounted for by some unexamined variable within modern diversity but this explanation is unlikely. In PCAs specimens from the three sites occupy regions of the first two PCs not occupied by modern turtles. Results of disparity comparisons also support the hypothesis that fossilized *T*. *carolina* occupy regions of morphospace not occupied by modern samples from across the range of *T*. *carolina* in the United States.

Some of the specimens significantly different from modern specimens may correspond to the extinct species *T*. *putnami*. The holotype of *T*. *putnami* is a nondiagnostic fragment of a left hypoplastron, and the carapace of the neotype was not available for analysis at the time of this study [27]. However, the neotype comes from the Haile 8A locality from which several other carapaces were analyzed [44]. Future analyses of the apomorphies of *T*. *putnami* and their application to the specimens assigned to the third morph in this study would help support or refute the interpretation that this morphology represents a distinct species, *T*. *putnami*. Rigorous documentation of morphological variation within *T*. *c*. *mexicana* and *T*. *c*. *yucatana* may provide additional insight. Revised average shell profiles of *T*. *yucatana* in particular bear some resemblance to specimens from Haile 8A in terms of the boxy, sharply sloping carapace profile, reduced flaring of the peripherals, and notable midline dorsal keel (Fig 49.1 of [81]).

Much about the fossil record of *T*. *carolina* remains unexplained. The novel morphology in the fossil record is not strictly associated with size or age. Specimens from Camelot, the geologically oldest site in the study, are not the most different from modern specimens if they are significantly different at all (Figs 11 and 12, Table 10). The best clustering model, *k* = 1, does not support the hypotheses that any part of the fossil record forms a species that is always discretely different from *T*. *carolina*. Some specimens included in this study were previously described as hybrids of *T*. *carolina* and *T*. *putnami* [22]. More work will be necessary to determine if hybridization or anagenetic evolution explain the presence of distinct morphology but the lack of discrete groups in the fossil record of *T*. *carolina*.

The most puzzling aspect of that record yet to be explained is the presence of large and small morphs of *T*. *carolina* discovered together at the same site. The members of each morph occupy non-overlapping carapace length ranges and those ranges are separated by at least 60 mm with no specimens of intermediate size (Fig 10). The presence of two sympatric species was previously hypothesized to explain this phenomenon at the Melbourne-Vero site pair, though the explanation was not universally accepted [21,29]. The results presented here eliminate alternative possibilities that sexual dimorphism or differences between juveniles and adults could explain the pattern. They also extend that pattern of sympatric morphs both spatially and temporally. The Irvingtonian Camelot site in South Carolina contains two morphs that cannot be attributed to allometric or sexually dimorphic variation. However, the large and small morphs at either site are not significantly different from each other in shape when the growth correction is applied. It is possible that the lack of significant shape difference could be a methodological artefact of comparing two groups with small sample sizes, but additional fossils are necessary to test that hypothesis. Regardless of cause, the presence of sympatric morphs is a novel pattern in the fossil record not seen in the modern species.

## Conclusion

This study contributes to the body of literature that assesses identification practices within a chelonian species [85,138,162]. Aspects of shell morphology have long been used as proxies for sex and presence or absence of reproductive maturity [29,163], but the features that are most applicable to the fossil record are those that have been least vetted [84,85]. In assessing all three previously proposed shell-based proxies for maturity, this study evaluates the means by which the modern biota and fossil record can be analyzed in a common framework. The assessment of the utility of shell-based proxies for sex provides additional quantitative evaluation of proxies often applied uncritically to the fossil record [29,30,158].

After evaluating the utility of those identification practices, the study builds on the common framework of carapace morphology to interpret the fossil record in light of documented, quantified patterns of variation in the modern biota. Of the five morphs previously proposed to make up the Pleistocene fossil record of *T*. *carolina*, two at best can be recognized in this study. Future work is necessary to evaluate the correct taxonomic names, evolutionary relationships, and possible intermediates of diagnosable entities in the fossil record.

Notably, the fossil record could only be interpreted after additional analyses of standing variation in the modern biota. The inability to recognize extant, intraspecific entities in the osteology of *T*. *carolina* stands in contrast to the identification of intraspecific units in the fossil record of marmots and shrews [1,20]. It is possible that intraspecific entities could be recognized in additional vertebrate taxa, but those possibilities can only be explored if variation is documented in the appropriate morphological system for each taxon [1,20,176]. That work is an important step in creating comparable datasets that can be used to test hypotheses of long-term evolution derived from the relatively short-term records contained within the modern biota.

## Supporting information

S1 File Specimens included in analyses of proxies of maturity. (XLSX)

S2 File Specimens included in geometric morphometric analyses. (XLSX)

S3 File Supporting information about the age of fossil sites reported in text. (DOCX)
