# Supplementary material for: Delineating modern variation from extinct morphology in the fossil record using shells of the Eastern Box Turtle (Terrapene carolina)
Source: PLoS One. 2018 Mar 7;13(3):e0193437. doi: 10.1371/journal.pone.0193437 (PMC5841793; doi:10.1371/journal.pone.0193437)
Supplement: S3 File — (DOCX) [file pone.0193437.s003.docx]

**S3 File. Supporting Information about the age of fossil sites reported in text.**

Grove’s Orange Midden is an archeological site in Florida with associated radiocarbon dates of 3.8–6.2 thousand years before present (ka) [1]. Fort Center, a second Floridian archeological site, was similarly dated to 0.45–2.8 ka [2,3]. Specimens were as *T. carolina* by the apomorphic combination of co-ossified shells with a prominent dorsal keel and the absence of a sutured connection between the carapace and plastron [4]. At Friesenhahn Cave in central Texas, radiocarbon dates of 17,800 ± 880 and 19,600 ± 170 years are associated with Level 3, from which the box turtle fossils of the site were recovered [5,6]. The Ardis site in South Carolina is associated with radiocarbon date of 18.5 ka and an upper bound on the age of the site estimated at 22 ka [7].

Vero and Melbourne are two latest Pleistocene sites discovered near each other in the early twentieth century on the eastern coast of Florida [8,9]. Fossils of humans and megafauna were recovered from both sites. Results of subsequent rare earth element analysis support the contemporaneity of the species [8–10]. In addition to similar faunas, the two sites also have similar lithologies and stratigraphic layers [8,9]. They are considered to be coeval in this study and are treated as a closely related site pair given their geographic proximity to each other. Devil’s Den is another Florida site in which humans and megafauna were contemporaneous [11]. Based on the first well-dated occurrence datum for humans in North America and the last occurrence data for Pleistocene megafauna in North America, the age of Vero, Melbourne, and Devil’s Den is bracketed between 12.7 and 9.5 ka [11,12].

At Haile 8A, the box turtles themselves were previously used as evidence for the age of the site [13,14]. Haile 8A was hypothesized to preserve a transitional sequence of fossils starting from *T. c. bauri* or *T. c. carolina* and going to *T. c. putnami* based on an increase in size, peripheral flaring, and development of a midline keel [14]. The subspecies identities were linked to ecological requirements, and considered evidence of a rising sea level [14,15]. An evaluation of that hypothesis is part of this study and therefore it would be circular to use it to justify an age for the site. Isotopic evidence from bulk and serial sampling of mammalian tooth enamel support a transitional climate without relying on conclusions from box turtles [13]. The lithology of the site, in which organic-rich lower beds transition to more lateritic beds, is also consistent with an environmental interpretation of rising sea levels [14].

The Haile 8A fauna contains *Bison* and extinct megafauna, biostratigraphically bracketing it within the Rancholabrean North American Land Mammal Age (NALMA) [15]. Reddick 1B, like Haile 8A, can be placed in the Rancholabrean North American Land Mammal Age based on the presence of *Bison* and megafauna [16]. The first well-dated appearance of *Bison* south of 55° N latitude is currently 160 ka, and the last appearance of Pleistocene megafauna is 9.5 ka, constraining the Rancholabrean as we currently understand it to between those dates [12].

Reddick 1B was previously further constrained to the Late Rancholabrean and Haile 8A to the Early Rancholabrean based on associated mammalian taxa [17]. The division between the proposed Early and Late Rancholabrean was marked by the replacement of *Bison latifrons* by *Bison bison antiquus* [12,18]. It has since been recognized that *B. latifrons* persists in the southeastern United States as late as 19.8 ka [19]. It is not clear if the Early and Late Rancholabrean have a much younger boundary than was previously thought or if the chronology of *Bison* is more complex than a simple replacement pattern even when the chronology is spatially limited to Florida [12]. Pending clarification of that uncertainty, Haile 8A and Reddick 1B are conservatively considered to be biostratigraphically Rancholabrean with no subdivisions. There are multiple transitions from glacial to interglacial stages within the Rancholabrean, and the age of either site cannot be further temporally constrained.

The numerical ages of Ingleside, a coastal site in Texas, are variably reported to be ~120ka, 30-25 ka, 80-10 ka, and 75-30 ka based on sequence stratigraphy, deep sea oxygen isotope records, and mammalian biostratigraphy [20–24]. The presence of *Bison* and megafauna at Ingleside constrains the site to the currently understood temporal boundaries of the Rancholabrean, like Haile 8A and Reddick 1B. The site was deposited during an interglacial interval based on faunal and stratigraphic evidence [25,26]. The only interglacial currently recognized during the Rancholabrean is MIS 5 [27]. The site has been proposed to be further restricted to MIS5e, currently dated to 130-120 ka, based on sequence stratigraphy [24,26].

The oldest site from which multiple, complete carapaces of *T. carolina* were available is the Camelot site in central South Carolina. The site was initially considered to be ~400 ka based on faunal equivalence to the Coleman 2A fauna in Florida and stratigraphic evidence [28]. Based on the occurrence of *Canis armbrusteri*, an index taxon for the Irvingtonian, and the absence of *Bison*, the site can at least be constrained to the Irvingtonian [12,28,29]. Additionally, the occurrence of *Didelphis* and the presence of specimens of *Smilodon* and *Megalonyx* with morphology intermediate between early Irvingtonian and Rancholabrean species all support a late Irvingtonian age, as was previously proposed [12,28,29]*.* The late Irvingtonian was is constrained by the Cudahy (middle Irvingtonian) Lava Creek B Ash, which was dated to 0.61 Ma, and the start of the Rancholabrean, which is dated to 160 ka as of this study [17].

Literature Cited

1. McGee RM, Wheeler RJ. Statigraphic excavations at Groves’ Orange Midden, Lake Monroe, Volusia County, Florida: methodology and results. The Florida Anthropologist. 1994;47: 333–349.

2. Pluckhahn TJ, Thompson VD. Integrating LiDAR data and conventional mapping of the Fort Center site in south-central Florida: A comparative approach. Journal of Field Archaeology. 2012;37: 289–301. doi:10.1179/0093469012Z.00000000026

3. Franz R, Quitmyer IR. A fossil and zooarchaeological history of the gopher tortoise (*Gopherus polyphemus*) in the southeastern United States. Bulletin of the Florida Museum of Natural History. 2005;45: 179–199.

4. Joyce WG, Petricevic A, Lyson TR, Czaplewski NJ. A new box turtle from the Miocene/Pliocene boundary (latest Hemphillian) of Oklahoma and a refined chronology of box turtle diversification. Journal of Paleontology. 2012;86: 177–190.

5. Milstead WW. Fossil turtles of Friesenhahn Cave, Texas, with the description of a new species of *Testudo*. Copeia. 1956;1956: 162–171.

6. Graham RW. Pleistocene and Holocene mammals, taphonomy, and paleoecology of the Friesenhahn Cave Local Fauna, Bexar County, Texas. PhD dissertation, The University of Texas at Austin. 1976.

7. Bentley CC, Knight JL, Knoll MA. The mammals of the Ardis Local Fauna (Late Pleistocene), Harleyville, South Carolina. Brimleyana. 1994;21: 1–35.

8. Gidley JW. Investigating evidence of early man in Florida. Smithsonian Miscellaneous Collections. 1927;78: 168–174.

9. Stewart TD. A reexamaniation of the human skeletal remains from Melbourne, Florida, with further data on the Vero skull. Smithsonian Miscellaneous Collections. 1946;

10. MacFadden BJ, Purdy BA, Church K, Stafford TW. Humans were contemporaneous with late Pleistocene mammals in Florida: evidence from rare earth elemental analyses. Journal of Vertebrate Paleontology. 2012;32: 708–716. doi:10.1080/02724634.2012.655639

11. Purdy BA, Rohlwing KM, MacFadden BJ. Devil’s Den, Florida: Rare Earth Element Analysis Indicates Contemporaneity of Humans and Latest Pleistocene Fauna. PaleoAmerica. 2015;1: 266–275. doi:10.1179/2055556315Z.00000000032

12. Bell CJ, Lundelius Jr EL, Barnosky AD, Graham RW, Lindsay EH, Ruez Jr DR, et al. The Blancan, Irvingtonian, and Rancholabrean mammal ages. In: Woodburne MO, editor. Late Cretaceous and Cenozoic Mammals of North America. New York: Columbia University Press; 2004. pp. 232–314.

13. Yann LT, DeSantis LRG. Effects of Pleistocene climates on local environments and dietary behavior of mammals in Florida. Palaeogeography, Palaeoclimatology, Palaeoecology. 2014;414: 370–381. doi:10.1016/j.palaeo.2014.09.020

14. Auffenberg W. Further notes on fossil box turtles of Florida. Copeia. 1967;1967: 319–325. doi:10.2307/1442120

15. Webb SD. Chronology of Florida Pleistocene mammals. In: Webb SD, editor. Pleistocene Mammals of Florida. Gainesville: The University Presses of Florida; 1974. pp. 5–31.

16. Gut HJ, Ray CE. The Pleistocene vertebrate fauna of Reddick, Florida. Quarterly Journal of the Florida Academy of Sciences. 1963;26: 315–328.

17. Morgan GS, Hulbert, Jr. RC. Overview of the geology and vertebrate biochronology of the Leisey Shell Pit Local Fauna, Hillsborough County, Florida. Morgan GS, Hulbert, Jr. RC, Webb SD, editors. Bulletin of the Florida Museum of Natural History. 1995;37 Pt. 1: 1–92.

18. Hulbert, Jr. RC, editor. The Fossil Vertebrates of Florida. University Press of Florida; 2001.

19. Patterson DB, Mead AJ, Bahn RA. New skeletal remains of *Mammuthus columbi* from Glynn County, Georgia with notes on their historical and paleoecological significance. Southeastern Naturalist. 2012;11: 163–172. doi:10.1656/058.011.0201

20. Fulton RJ, Prest VK. Introduction: The Laurentide ice sheet and its significance. Géographie physique et Quaternaire. 1987;41: 181–186. doi:10.7202/032676ar

21. Baskin JA. Early Pliocene horses from late Pleistocene fluvial deposits, Gulf Coastal Plain, South Texas. Journal of Paleontology. 1991; 995–1006.

22. Goodwin HT. Pliocene-Pleistocene biogeographic history of prairie dogs, Genus *Cynomys* (Sciuridae). Journal of Mammalogy. 1995;76: 100–122. doi:10.2307/1382319

23. Koch P. The effects of late Quaternary climate and pCO2 change on C4 plant abundance in the south-central United States. Palaeogeography, Palaeoclimatology, Palaeoecology. 2004;207: 331–357. doi:10.1016/j.palaeo.2003.09.034

24. Yann LT, DeSantis LRG, Koch PL, Lundelius EL. Dietary ecology of Pleistocene camelids: Influences of climate, environment, and sympatric taxa. Palaeogeography, Palaeoclimatology, Palaeoecology. 2016; doi:10.1016/j.palaeo.2016.08.036

25. Lundelius, Jr EL. Fossil vertebrates from the Late Pleistocene Ingleside Fauna, San Patricio County, Texas. Bureau of Economic Geology Report of Investigations. 1972;77: 1–74.

26. Otvos EG, Howat WE. South Texas Ingleside barrier; coastal sediment cycles and vertebrate fauna. Late Pleistocene stratigraphy revised. Transactions of the Gulf Coast Association of Geological Societies. 1996;46: 333–344.

27. Railsback LB, Gibbard PL, Head MJ, Voarintsoa NRG, Toucanne S. An optimized scheme of lettered marine isotope substages for the last 1.0 million years, and the climatostratigraphic nature of isotope stages and substages. Quaternary Science Reviews. 2015;111: 94–106. doi:10.1016/j.quascirev.2015.01.012

28. Kohn MJ, McKay MP, Knight JL. Dining in the Pleistocene—Who’s on the menu? Geology. 2005;33: 649–652. doi:10.1130/G21476.1

29. Fields SE. The ground sloth Megalonyx (Xenarthra, Megalonychidae) from the Pleistocene (late Irvingtonian) Camelot local fauna, Dorchester County, South Carolina. Philadelphia: American Philosophical Society; 2010.
